# What constitutes a palliative care need in people with serious illnesses across Africa? A mixed-methods systematic review of the concept and evidence

**DOI:** 10.1177/02692163211008784

**Published:** 2021-04-16

**Authors:** Oladayo A Afolabi, Kennedy Nkhoma, Matthew Maddocks, Richard Harding

**Affiliations:** 1 Cicely Saunders Institute of Palliative Care, Policy and Rehabilitation, Florence Nightingale Faculty of Nursing, Midwifery and Palliative Care, King’s College London, London, UK; 2 Department of Nursing Science, University of Maiduguri, Maiduguri, Nigeria

**Keywords:** Palliative care, needs assessment, Africa, terminally ill

## Abstract

**Background:**

Clarity on what constitutes a palliative care need is essential to ensure that health systems and clinical services deliver an appropriate response within Universal Health Coverage.

**Aim:**

To synthesise primary evidence from Africa for palliative care needs among patients and families with serious illness.

**Design:**

We conducted a mixed methods systematic review with sequential synthesis design. The protocol was registered with PROSPERO (CRD42019136606) and included studies were quality assessed using Mixed Method Appraisal Tool.

**Data sources:**

Six global literature databases and Three Africa-specific databases were searched up to October 2020 for terms related to palliative care, serious illnesses and Africa. Palliative care need was defined as multidimensional problems, symptoms, distress and concerns which can benefit from palliative care.

**Results:**

Of 7810 papers screened, 159 papers met eligibility criteria. Palliative care needs were mostly described amongst patients with HIV/AIDS (*n* = 99 studies) or cancer (*n* = 59), from East (*n* = 72) and Southern (*n* = 89) Africa. Context-specific palliative care needs included managing pregnancy and breastfeeding, preventing infection transmission (physical); health literacy needs, worry about medical bills (psychological); isolation and stigma, overwhelmed families needing a break, struggling to pay children’s school fees and selling assets (social and practical needs); and rites associated with cultural and religious beliefs (spiritual).

**Conclusions:**

Palliative care assessment and care must reflect the context-driven specific needs of patients and families in Africa, in line with the novel framework. Health literacy is a crucial need in this context that must be met to ensure that the benefits of palliative care can be achieved at the patient-level.

**What is already known about this topic?**

- People with serious illness in low-and-middle-income countries constitute 80% of the population with palliative care needs globally.
- The construct of ‘total pain’ which underpins palliative care evolved in high-income western societies with social welfare provision.
- Universal Health Coverage states that access to healthcare (including palliative care) should not put people out of pocket.

**What this paper adds?**

- An evidenced-based framework for systematically understanding the specific palliative care needs of patients with serious illnesses in Africa built from the evidence for specific needs in African contexts.
- Social, practical support, information and financial needs cannot be neglected in the estimation of needs and the design of palliative care models in Africa.
- This contextual understanding is needed to ensure that the global drive to Universal Health Coverage is achievable.

**Implications for practice, theory and policy**

- To alleviate suffering within Universal Health Coverage in the African context we must look beyond physical and psychological needs to address social and spiritual needs.
- The design and testing of models of delivering palliative care must be guided by the proposed African-specific framework of needs, and expand beyond HIV and cancer to focus on other conditions which will drive future palliative care needs in Africa.
- The proposed framework should also inform clinical multidimensional needs assessment of patients in Africa that encompasses context-driven needs.

## Background

Palliative care improves patient-reported outcomes, patient and caregiver satisfaction and quality of life,^1–3^ and can be effective and cost-effective in low-and-middle-income countries.^4^ Palliative care is an essential health service within Universal Health Coverage.^5^

The term ‘need’ has been conceptualised differently across research disciplines (sociology, philosophy, economics and health).^6^ Within healthcare, needs are defined at two levels, individual healthcare needs and community/population healthcare needs.^6^ While population-level needs help to guide the allocation of resources and designing of services, individual-level needs are the primary targets of different health interventions and provide insights which are useful in developing and refining practice.

Three core conceptualisations of needs inform population-level palliative care needs assessment.^7^ Maslow’s hierarchy of needs conceptualises human needs at the individual level as universal and essential triggers for innate human motivation^8^; Bradshaw conceptualised needs at the individual, service provision and population-level depending on how and by whom the need for social services is defined^9^; while Steven and Raftery’s definition of healthcare need as the ‘capacity to benefit’ from something^10^ has been majorly applied in service provision. The understanding of individual-level needs within palliative care is underpinned by the holistic and multidimensional approach to the patient and family’s suffering as a result of their unmet physical, psychological, social and spiritual needs.^11^ This is reflected in the WHO’s palliative care definition.^12^

Palliative care interventions must be person-centred and targeted at patients’ specific needs. Palliative care and its underpinning concepts of ‘total pain’ and ‘holistic care’^11,13–15^ were shaped by early development and adoption in high-income countries with welfare states, cost-protective healthcare services and developed public health systems. Achievement of palliative care goals within Universal Health Coverage strategy requires an understanding of how palliative care needs in low-and-middle-income countries may differ from high-income countries within the framework of physical, psychological, social and spiritual domains.^16,17^ This demands clear articulation of what constitute palliative care needs of patients within different cultural and socioeconomic contexts.

Within Africa lie 40% of the world’s countries classified as low or middle income by the World Bank (the highest percentage from any continent), with a population of 1.3 billion people. This review aimed to synthesise the evidence of what constitutes the palliative care needs of patients with serious illness and their families in Africa.

## Methods

The study’s objectives were to: (1) map and synthesise the evidence on needs of people with serious illness and their families in Africa; (2) critically appraise this evidence on needs in Africa based on the WHO’s definition of palliative care.

### Design and registration

This mixed-methods systematic review was conducted using sequential synthesis design.^18^ The design involved a framework synthesis of qualitative data^19,20^ after which the emergent framework was used to analyse the quantitative data. This involved both aggregative and configurative approach to interpret and understand the evidence in order to clarify the concept.^18,20^ This was reported following the Preferred Reporting Items for Systematic Reviews and Meta-Analyses (PRISMA) statement^21^ and Enhancing Transparency in Reporting the Synthesis of Qualitative Research (ENTREQ) guidelines.^22^ The protocol was registered with PROSPERO (CRD42019136606).

### Search strategy

Search terms for the main concepts of the review question ([Palliative care OR related terms] AND [Serious illnesses OR Related terms] AND [Africa OR related terms]) (Supplemental File 2) were used. Subject-headings and keywords were searched in nine electronic bibliographic databases (MEDLINE, EMBASE, PsycINFO, Global Health, SCOPUS, CINAHL and three databases focused on Africa-Africabib.org, Catalogue of the African studies centre at the University of Leiden and African Journal online- from inception to 16 October 2020. References of included studies were hand-searched for additional studies that met inclusion criteria.

### Inclusion and exclusion criteria

We adopted the WHO’ definition that palliative care is applicable early in the course of illness and, in conjunction with other therapies that are intended to prolong life. Therefore, we included studies at all phases of the selected illnesses. This was considered practical as it was difficult to precisely identify the phase of illness from majority of the studies. To identify life-threatening illnesses for this study, we adopted the definition of serious illnesses which has been widely used in global advocacy.^23^ Serious illnesses were defined as chronic conditions which demonstrate palliative care needs, and cause mortality and/or hospitalisation in the year before death. These included cancers, dementia, organ failure (heart, lung, renal and liver) and other diseases (such as HIV, stroke and neurological diseases).^24,25^

A preliminary search revealed that the term ‘palliative care needs’ was seldom used within the literature from Africa. Rather terms such as multidimensional distress or problems, symptom distress or burden, palliative care problems and physical and psychosocial symptoms or concerns were used to describe individual-level needs amenable to palliative care. Therefore, we defined palliative care needs as individual-level problems, symptoms, distress and concerns in the physical, psychological, social and spiritual domains of life which have the capacity to benefit from a palliative care intervention. In order to avoid losing studies which addressed this definition of palliative care needs but which did not use the term, the concept of need was not used in our search terms. However, we applied it in the selection of studies for inclusion (criteria are detailed Table 1). Studies in English and French languages were included. Randomised controlled trial designs were excluded as they are designed and conducted under tightly controlled conditions and specifications; thus, they may not adequately reflect the individual-level palliative care needs of the patients.

**Table 1.** Inclusion and exclusion criteria.

Inclusion criteria	Exclusion criteria
• Studies conducted in any African country. • Population: Adults (⩾15 years old) and their family carers (anyone ⩾15 years involved in providing care for the patient with a familial relationship with them.) • Studies at any phase of a serious illness defined as chronic conditions which demonstrate palliative care needs, constitute underlying and contributory causes of mortality and account for hospitalisation in the year before death. These included cancers, dementia, organ failure (heart, lung, renal and liver) and other diseases (such as HIV, stroke and neurological diseases) • Only empirical studies • Studies reporting palliative care needs, (i.e. individual-level problems, symptoms, distress and concerns in the physical, psychological, social and spiritual domains of life which have the capacity to benefit from palliative care as defined by the WHO)^12^	• Studies in African Americans or African immigrants outside of Africa • Multinational studies involving countries outside of Africa unless data is appropriately disaggregated • Studies in children or individuals ⩽15 years old and their family carers • Children family carers for adult patients • Acute infectious diseases • Epidemiological studies that only focused on a statistical projection or estimation of the prevalence of certain serious illnesses as proxies for describing palliative care need • Designs: ○ RCTs ○ Case studies ○ Validation studies of measurement tools • Studies not focusing on palliative care needs, for example studies only reporting clinical profile or medical management of serious illness, survival analysis, surgical pathways for cancer and general perception • Editorials, opinions and Commentaries

### Study selection

All search results were exported to Endnote version X9 where de-duplication was conducted, and references were managed by the first reviewer (OA). Titles and abstracts of all articles were also screened by OA to ascertain their relevance and whether the inclusion or exclusion criteria were met. A second reviewer (KN) double-checked all included articles. Any article for which inclusion was unclear was also discussed with KN and if necessary adjudicated by third reviewers (MM and RH).

### Data extraction

OA extracted relevant data into a common table: author and year of study, study design, aim, setting and country of the study, diagnoses, domains of needs reported in line with the WHO definition, themes on domains of needs and factors reported to be related to or associated with palliative care needs. Data extraction was reviewed by KN, MM and RH.

### Quality assessment

OA and KN appraised study quality using Mixed Methods Appraisal Tool (MMAT)^26^ which has two screening questions and other criteria to rigorously appraise different study designs within five methodological domain: (1) qualitative studies; (2) randomised controlled quantitative studies; (3) non-randomised quantitative studies; (4) observational descriptive quantitative studies; and (5) an additional set of criteria for mixed methods research studies. For mixed methods studies, study components were first assessed separately as above, followed by the additional mixed-methods quality criteria. Thus, for studies that fulfilled the first four methodological domains, the total obtainable appraisal score was 6 while for the mixed method, the total obtainable score was 13.

### Analysis and synthesis

We conducted narrative synthesis using framework synthesis approach. We summarised the evidence based on countries where the research was conducted and the population. The synthesis was conducted in three stages.

First, best-fit framework synthesis^19^ was used to appraise the qualitative data. The method provides a means to test, reinforce and build on existing models and frameworks.^19^ This involved a combination of deductively coding against apriori themes from the identified model and inductively coding new themes to identify areas not currently addressed in the current model. The widely used WHO definition of palliative care and guide for the implementation of palliative care within primary healthcare^12,27^ was adopted as the apriori framework for the synthesis using deductive analysis. The definition highlights that patients and family caregivers are the unit of care in palliative care. It also stated the problems benefitting from palliative care (i.e. palliative care needs) in patients (physical, psychological, social and spiritual) and family carers (psychological, social and spiritual).

OA and KN iteratively coded data on palliative care needs through secondary thematic synthesis. In some instances, we semantically interpreted^28^ palliative care needs from the author explanations and participant quotes in the studies’ result, and arranged the themes together into overarching themes using existing domains.^28^ Where applicable, new themes were inductively generated while testing the fit of the coded domains of palliative care needs to the WHO definition. MM and RH verified the data analysis.

Secondly, the new framework generated from our synthesis was then used to appraise all included quantitative and qualitative studies for reporting of palliative care need. We used truth tables^29^ to identify and aggregate the presence or absence of the domains of palliative care needs within the different studies guided by the new framework. Truth tables are a form of matrix in which all possible combinations of conditions (e.g. participant and intervention characteristics) are cross-tabulated against each study. We then assigned binary format (yes or no) when a domain of need is present or absent within a study respectively.

Lastly, we created a cross-tabulation matrix to understand the relationships across different domains of palliative care needs and the factors associated with and related to palliative care needs as reported in the studies. We grouped the associated factors based on Person-Health-Environment-Treatment framework.^30^ Person refers to factors related to the patient or the family which are the units of care. Health refers to the factors related to the changes in health status, the markers of illness or disease condition; and factors internal and external to the patients and families which affect them were coded under environment. Treatment-related factors were factors which address the care being given to the patients and how this was done.

## Results

### Included studies

**Figure 1.**
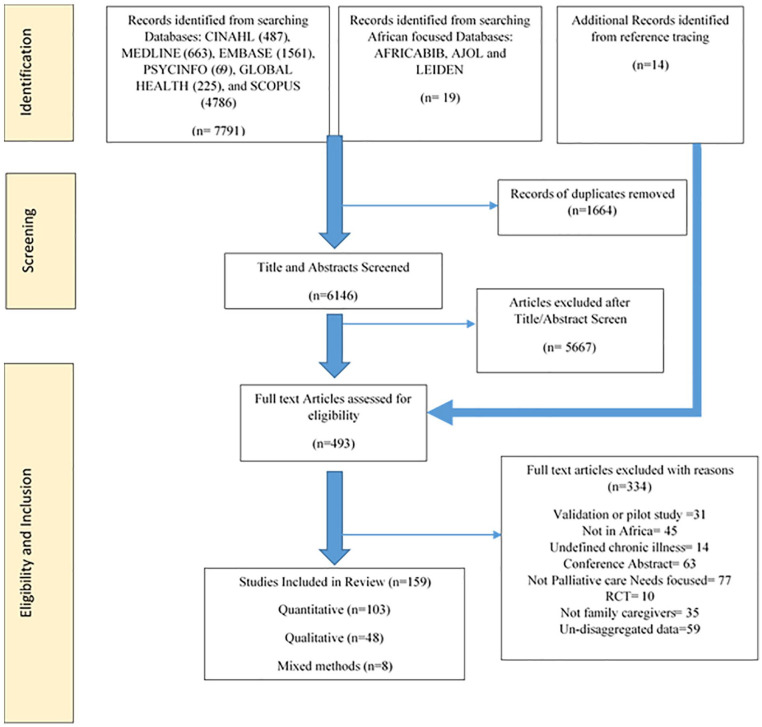
PRISMA flow chart of study selection.

The electronic database searches yielded 6164 results after deduplication. Of these, we reviewed the full text of 479 articles and identified additional 14 papers through searching reference lists. One hundred and fifty-nine papers met the eligibility criteria for the synthesis. The articles included *n* = 103 (65%) quantitative, *n* = 48 (30%) qualitative and *n* = 8 (5%) mixed methods studies. The PRISMA flow chart^21^ details the stages of inclusion and exclusion (Figure 1). Details of all included studies are presented in the Supplementary File 1.

### Quality appraisal

Majority of the articles met most of the quality criteria (Supplemental File 1). Majority of the single design studies met between 4 and 6 criteria with a median score of 5 (range 2–6). Most of the qualitative studies (*n* = 29) did not meet the criteria on reflexivity, that is whether appropriate consideration was given to how the findings relate to the researcher’s influence. Only *n* = 14 quantitative studies fully met the criteria on whether the sample used was representative of the population. For the *n* = 8 mixed methods articles, the majority of the quality criteria were met except for the criteria on whether appropriate considerations were given to the limitations associated with the integration of the findings such as the divergence of qualitative and quantitative data in triangulation design. We retained all studies in the analysis.

### Context

**Figure 2.**
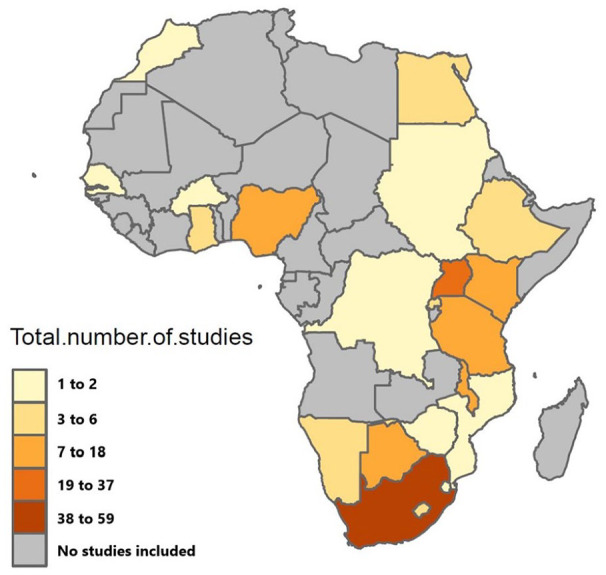
Distribution of studies by country.

The included studies (*n* = 159) were from 22 African countries (Figure 2). The majority of the studies were conducted in countries in Southern (*n* = 89) and East Africa (*n* = 72), with very few studies from Central (*n* = 1), West (*n* = 18) and North African countries (*n* = 6) meeting inclusion in this review.

**Figure 3.**
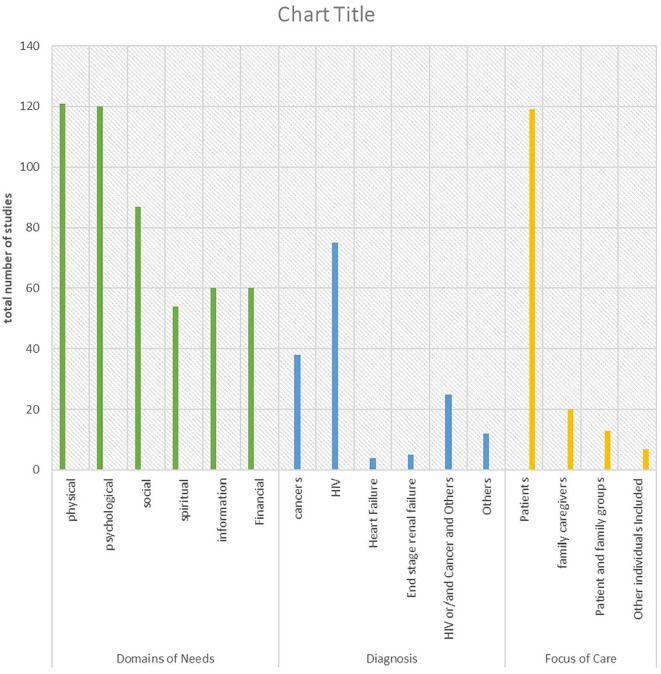
Mapping of evidence according to the domain of needs, diagnosis and focus of care.

The number of studies based on diagnosis is shown in Figure 3. The majority of studies reported on patients with HIV/AIDS (*n* = 75), cancer (*n* = 38); *n* = 25 studies had aggregated data on HIV/AIDS and/or cancer and other terminal illnesses (including liver failure, liver cirrhosis, renal failure, heart failure, cardiomyopathies, Kaposi sarcoma, motor neuron disease, stroke, tuberculosis, peripheral neuropathy, chronic obstructive pulmonary disease (COPD), SLE, MS, Korsakoff’s syndrome, cardiomyopathy, subarachnoid haemorrhage, anal fissure, paraplegia and other unspecified terminal illness), heart failure (*n* = 4), end-stage renal failure (*n* = 5) and *n* = 12 studies with aggregated data-focused entirely on other serious illnesses (including Parkinson’s disease, tuberculosis, chronic liver diseases, COPD and other unspecified terminal illness).

A total of *N* = 119 (74.8%) studies were conducted with patients only, 20 (12.6%) studies with families only, 13 (8.2%) with both patients and family caregivers, while 7 (4.4%) included health care workers or volunteer caregivers (but data for patients and/ or family carers were not adequately disaggregated for our analysis).

### Domains of palliative care need reported

In addition to the domains within the WHO definition, our inductive analysis shows contexts-specific subthemes resulting in a new Africa-specific framework for domains of palliative care needs and the specific needs reported under each domain (Table 2).

**Table 2.** The framework of palliative care needs including added domains.

Patient	Families
**Physical needs**	
Pain and other Physical Symptoms^31–41^ Nutritional Support^36,41–46^ Functional limitations (Inability to conduct activities of daily living)^35,43,44,47–49^ Wounds and wound odours^33,44^ Libido problems and sexual needs^31,35,50–53^ Reproduction (Pregnancy and breastfeeding)^40,51,44,49,54–56^ Cognitive and behavioural problems^57^	Physical symptoms/ physical health suffering as a result of providing physical care^58^: fatigue,^59^ exhaustion,^45^ sleeplessness,^59^ back pain,^60,61^ chest and shoulder pain^61^ Neglecting own self and personal symptoms^45,58,60,62^ Protection from communicable infections^62–65^
**Psychological needs**	
**Emotional needs** Psychological symptoms (depression, anxiety, internalised stigma)^31,32,34,35,38–41,56,63,66,67^ Lingering fear of death and Struggling to survive^41,43,53,62^ Worry (about the future, about prolonged illness and emaciation, about finances and affordability of medical bills)^35,36,50–52,54,56,68,69^ Body image and Self-esteem problems^41,50,51,62,70^ Dignity, loss of independence, loss of control^36,37,41,43,48,49,53,56,69^ Failed coping (acceptance with hopelessness), denial^31,33,36,40,49,54,71^ Somatised symptoms – depression and anxiety as sleeplessness^57^ Poor emotional support in hospital^39,40,66,72,73^ Anticipatory grieving^56,66,70,74^	Burnout^45,61^ Psychological symptoms (Anxiety, worries)^36,55,59,61,63^ Emotional rollercoaster of Vulnerability, Helplessness, hopelessness and hope^59,63,72,75–77^ Vicarious humiliation and shame^35,48,59,61,78,79^ Uncontrolled anticipatory grieving^32,60,61^ Not feeling prepared for impending death^59,56,76,80^ Need for ongoing motivation^56^ Coping through silence and denial^76^ Struggling to provide emotional support when also in need of emotional support^59^
**Information needs** Poor communication from healthcare professionals^40,43,55,71,81^ Lack of clear information in care coordination^44,82^ Need to know disease causation and signs and symptoms to expect^34,35,38,51,55,56,68,71,73,81^ Need to know how to care for self^35,43,54,73^ Need to know what next and disease course^55,71^ Need to know what treatment and support options are available^35,55,81^ Fear that palliative care will hasten their death^52^ Associating causation to witchcraft^31,33,50,68,74,81^ Delayed care seeking due to ignorance^33,47,53^ Things left unsaid in breaking the news of disease^71^ Support groups as sources of information^77^ Shielding self from information load^55,83^	Poor communication from Healthcare professionals^60,72,80,84,85^ Need to know disease causation and disease course^34,37,39,56,78,85,86^ Need to know how to protect self from contacting communicable infection^61,63,65,78^ Need training on providing health-related care for the patient at home^32,37,39,44,48,55,58,63,80,85,87,88^ Anxiety about handling the dying patient and dead body^37,39^ Need to know what options are available^37^ Secrecy and lack of disclosure around communicable diseases^56,63,75,76^ Initiating and managing end of life discussions with sick relative^37,84^ Unrealistic expectation due to poor information^54,75,80,83^ Not engaged in decision making^80^ Anger from lack of understanding^60,86^ Confusion and Uncertainty due to lack of information^37,58,78^ Silence and reluctance to talk about disease and prognosis^76,84^
**Social needs**	
**Relationship needs** Isolation and loneliness^33,40,41,44,50,51,53,56,66,74,77^ Deteriorating social and family networks^49,56,66,67,69,77^ Discrimination and stigma due to illness cause (promiscuity based or mysterious)^35,40,51,53,56,62,67,77^ Cultural interpretations of problems^45^ Libido problems, Sexual needs and intimacy^35,50,51,52,67^ Fear of disclosure^52,53,56,71^ Strain on relationship with family and friends^31,48,49,52,54^ Gender-specific impacts on women (exacerbated domestic violence)^67^	Restricted social networks^60,79^ Restricted social participation^59,61,76^ Coping with complex family dynamics and Disintegrating extended family^45,59^ Homecare is isolating^35,45,59,61,63,67,76^ Stigma^45,76,78^ Managing role reversal^45,79,86^ Caregiving strengthening family relationship^60^
**Practical support needs** Inability to fulfil social roles Decreasing support systems^36^ Childcare^36,56,66^ Need for support with shopping^77^ Practical support with care when bedridden^36,48,77^ Planning for children after their own death^37,43,56,69^	Relief/Respite from and support in caring^32,36,45,48,58,60,61,76,80,82,85^ Feeling overwhelmed and Needing a break^45,58–60,67,87^ Taking time off school and work^36,43,45,60,66,82^ Practical support in providing personal care where culturally inappropriate^79^ Restricted ambitions^60^ Managing fragmented care coordination^87^ Clinical supplies for care^63,75,85^
**Financial needs** Inability to earn income or unemployment^31,32,36,41,51,66^ Paying medical bills^31,36,39,43,49,50,53,54,66,71,74,82,86^ Cost of medications^31,37,38,44,49,54,77^ Securing transportation to appointments^32,40,44,48,49,53,66,68,87^ Paying children’s school fee^31,37,39,40,44,46,54,62,87^ Selling Assets^37,66,82^ Food insecurity and struggles^32,40,41,45,48,49,53,55,61,76,77,87^ Borrowing to pay for treatment^66,76^ Housing inconvenient for home-based care^32,40,43,46,65,77^ Becoming a financial burden^37,39,43,56,67,69^	Taking time off work and school^36,43,60,67,82^ Loss of income and assets^35,45,56,61,67^ Cost of medications^36,72,75,85^ Lack of enough finances to care for sick relative and family^32,35,36,40,46,58,59,61,76,85–87^ Economic drain of caregiving^63,88^ Loss of financial support of sick child^61^ Taking on patient’s financial responsibility, for example Paying orphaned children’s school fee^32,59,61,62,85^
**Spiritual needs**	
Finding meaning and spiritual purpose of illness^33,34,37,43,48,54,56,70,81,83^ Not feeling at peace^37,48^ Questioning God and anger at God^33,43,70,74^ Associating causation to witchcraft^31,33,38,47,50,68,74,81,83^ Guilt in reconciling causation, for example associating cancer as a punishment for sin^33,38,41,47,50,66,81^ Finding hope, using faith and Praying^31,33,36,38–41,54,66,80,83^ Cultural and Religious values and rites^31,48,69,74^ Dying without lineage propagation^33,38,40,42^ Dying without attaining life goals^43^	Seeking to make sense of the disease^37,45,86^ Cultural beliefs with spiritual implications, for example Feeling cursed due to taboos around performing culturally inappropriate care^37,60,79^ Vicarious suffering^72,75,80^ Uncertainty life, purpose and future Praying for healing and Finding comfort in God and help from ancestors^45,72,86^ Consulting witchdoctor^62^ Religious perpetuation of stigma^76^ Elderly carers feeling cursed as their children die before them^61^ Finding meaning in caregiving^88^

Figure 3 shows the distribution of the domains of palliative care needs reported across all included studies. Needs in the physical domain (*n* = 121 studies) and psychological domain (*n* = 120 studies) were the most commonly reported. Information needs were only explored and reported in about a third of the included studies.^2,16,31–38,42–45,47,48,50–55,58–60,63,64,66,68,71–78,80–87,89–104^

### The Africa-specific framework of palliative care needs

Both common and distinct needs were identified for patient and families.

#### Physical domain

Physical needs were reported by both patients and families. Physical needs in patients were highlighted in terms of pain and other physical symptoms and functional limitations in activities of daily living accompanying the illnesses. The need for nutritional support^36,41–46^ was also highlighted as cachexia and emaciation from the disease process are influenced by financial burden and food insecurity which compromises nutritional intake. An interesting aspect of physical need highlighted from a study is the need for reproductive care.^40,42,44,49,54–56^ Participants wanted to know how to conceive, deliver, breastfeed and take care of their babies even while they are suffering from a serious illness.

Family caregivers were developing physical symptoms such as fatigue, exhaustion, insomnia, backache, back pain, chest pain and shoulder pain^45,58–61^ due to their caring activities. They often neglected their physical symptoms^45,58,60,62^ while prioritising the patients’ symptoms, thus leading to worsening of their physical ailments. Due to poor communication from healthcare professionals about the contagious nature of the illness their sick relatives and the family caregivers’ caring disposition, family caregivers became infected by the patient’s disease as necessary precautions were not being taken.^62–65^

#### Psychological domain

##### Emotional needs

Most papers reported the psychological domain of needs; patients highlighted patterns of psychological symptoms such as worries, depression and anxiety.^31,32,34,35,38–41,56,63,66,67^ Psychological problems related to internalised stigma which are associated with communicable diseases and their perceived causes were also reported. Participants with HIV/AIDS felt isolated because others considered their sickness a result of their past bad lifestyle. The studies reported that people living with serious illness and their families felt devalued and lost dignity as they were being avoided when trying to borrow money, and as they become a financial burden on others. This further complicates the psychological burden of living with a serious illness. While suffering from these, participants highlighted the lack of emotional support from Healthcare professionals in the hospitals.^39,40,66,72,73^ Also, worries, anxieties and depression interact with other issues such as financial problems, uncertainties from lack of information and guilt from seeing disease as a punishment.^33,35,36,38,41,47,50–52,54,56,66,68,69,81^

In families, pervasive patterns of burnout and exhaustion,^45,61^ psychological symptoms (such as anxiety, worries),^36,55,59,61,63^ and helplessness and hopelessness^59,63,72,75–77^ were reported. Some studies found that family caregivers experienced vicarious humiliation and shame^35,48,59,61,78,79^ from the role reversals that take place as children care for parents or elderly parents caring for their adult children. This is also due to the stigma associated with the patient’s illness. A pattern of uncontrolled anticipatory grieving^32,60,61^ and feeling unprepared for impending death^56,59,76,80^ was also reported as family caregivers whose lives have become all about caring for the patient look forward to future uncertainties and the inevitable impending loss without support.

##### Information needs

Information needs were considered important to separate as a theme under the psychological domain of need, as themes were coded showing the influence of cultural and spiritual schemas of illness causation^31,33,86^ on the attitudes of participants to their signs and symptoms and healthcare-seeking.^33,47,53^ This led to complications which increased intensity of need. Also evident from the studies was a pervasiveness of mystery around serious illness due to lack of information which unnecessarily heightens worries, feelings of uncertainty, feelings of guilt and shame and lack of trust in the healthcare professionals.^37,58,78^ Participants highlighted themes around poor communication and lack of clear information from Healthcare professionals in which important information about the illness was left unsaid.^40,43,55,71,81^ Results also highlighted the participants’ need to know concerning disease causation, signs and symptoms to expect, self-care, disease progression and prognosis and available options of care.^34,35,38,43,51,54–56,68,71,73,81^

The studies also reported information needs demonstrated by family members. While some family members embraced the secrecy and lack of disclosure around the illness affecting their loved one,^56,63,75,76^ others voiced their apprehension and anger about the silence of the healthcare professionals and the patient, the reluctance to talk about disease and prognosis and the lack of engagement in decision making regarding care.^76,80,86^ They expressed their need to know the disease causation and disease course^34,86^ and how to protect self from contacting communicable infection.^61,63,65,78^ Studies also show that family caregivers voiced their anger at the lack of information from Healthcare professionals which make them appear foolish in their caring choices. Two studies reported the struggles of family members with providing care they are not trained to do for their patients at home.^32,37,39,44,48,55,58,63,80,85,87,88^ Another area of information need emphasised in one study is the need for information on initiating and managing end of life discussions with sick relative.^37,84^

#### Social domain

The social domain comprised of three separate themes: relationship needs, financial needs and practical support needs.

##### Relationship needs

Isolation and loneliness^33,40,41,44,50,51,53,56,66,74,77^ were the most reported social needs across all conditions. Participants described facing their illness or caring for their family members alone with little support available. Many of the studies also reported that many problems had cultural interpretations^45^ which enhances the proliferation of stigma^35,40,51,53,56,62,67,77^ thus limiting social networks and support for individuals. However, participants in some studies reported having their families as a strong source of social and emotional support^56,71^ while others highlighted the support from religious affiliations which helped to inspire hope.^33^ As patients become increasingly incapacitated by their conditions, they become unable to fulfil their normal roles and this creates tension as individuals struggle with role reversals, loss of control and loss of independence.^42^ Participants also reported problems with libido, meeting partner’s sexual and intimacy needs^35,50–52,67^ and how these threaten their relationships and lead to sexual violence.

A pattern of deteriorating social networks^49,56,60,66,67,69,77,79^ and social participation^59,61,76^ was shown in some of the studies reviewed. Patients highlighted having to deal with illness alone as families now live farther away. Family members from the studies also revealed the disintegration of the extended family^45,59^ which is usually a major source of support.

##### Financial needs

Studies reported the challenges patients and families face in paying already highly subsidised medical bills,^31,36,39,43,49,50,53,54,66,71,74,82,86^ balancing cost of medication^31,37,38,44,49,54,77^ with feeding while facing daily struggles with food insecurity.^32,40,41,45,48,49,53,55,61,76,77,87^ The problem of access which is already widespread due to limited services is further compounded by the financial burden of securing transportation to appointments.^32,40,44,48,49,53,66,68,87^ As reported in the studies, shame and loss of agency accompanies becoming a financial burden on others^37,39,43,56,67,69^ and borrowing to pay for treatment.^66,76^ The propagation of poverty which occurs as assets are being sold^37,66,82^ and money meant to pay for children school fee^31,37,39,40,44,46,54,62,87^ are being spent on managing serious illness was also highlighted. This is coupled with the inability of the sick individual to engage in economic activity to provide for the family. Six studies also reported on poor housing condition which are inadequate for home-based care and need for housing for patients who cannot afford rent any longer because of the financial burden of illness.^32,40,43,46,65,77^

Additionally, studies highlighted some other unique financial challenges which family caregivers face such as taking time off work and school^36,43,60,67,82^ to provide care. This often means loss of income^35,45,56,61,67^ for the individual and further contribute to lack of enough finances to care for sick relative.^32,35,36,40,46,58,59,61,76,85–87^ One study highlighted the need for support with clinical supplies to enable them to safely care for their loved ones at home.^85^

##### Practical support needs

As reported in the studies, social needs in families often overlap with the need for practical support in providing care for the patients. Family caregivers highlighted the isolating nature of homecare.^63^ They detailed how they usually take time off work, school and other social activities^36,43,45,60,66,82^ and end up with restricted social networks, restricted social participation and hindered personal ambitions.^60^ These underscore the need for often unavailable respite, break or support in caring for their sick family members.^32,36,45,48,58,60,61,76,80,82,85^ This meant family caregivers try to manage feeling overburdened and overwhelmed.^45,58–60,67,87^ In line with this, family members highlighted the extra burden on their limited finances and social participation caused by having to coordinate care for the sick patient within fragmented health systems. Another study highlighted the uncomfortable and culturally inappropriate necessity of personal care^79^ in some circumstances such as providing personal care for a sick parent of the opposite sex.

#### Spiritual domain

Themes from studies reviewed highlighted the struggles of patients and family caregivers in finding the meaning and spiritual purpose of the illness.^33,34,37,43,48,54,56,70,81,83^ This often leads to questioning God^33,43,70,74^ and feelings of guilt in reconciling causation, for eample associating cancer as a punishment for sin.^33,38,41,47,50,66,81^ Studies reported the underlying belief in spiritual causes of illness which propagates the association of causation to witchcraft.^31,33,38,47,50,68,74,81,83^ Such association may lead to spiritual distress and influence whether individuals feel at peace or not as they try to reconcile who they have offended to deserve their illness.

The studies highlighted the intertwined perception of spirituality and religion in Africa. The use of religion as a coping mechanism from spiritual distress was also reported in some studies. Patients described finding hope, using faith and praying^31,33,36,38–40,54,66,80,83^ to resolve worries and anxieties. The importance of religious values and rites^31,48,69,74^ and existential distress around dying without having a child for lineage propagation^42,33,38,40^ were also reported.

Family members highlighted the spiritual implications of cultural beliefs which aggravates spiritual distress. For example, families describe the taboos around seeing their parents nakedness and touching their parents’ perineal areas and cultural inappropriateness of the care which makes them feel cursed.^37,60,79^ One study described how their religion that is meant to be a source of support in their difficult times sometimes become a source of perpetuating of stigma.^76^ This leaves them feeling disorientated with the religion in which they seek solace and aggravates spiritual distress.

##### Factors associated with palliative care needs

Based on our synthesis of the quantitative studies, Table 3 shows the different factors associated with or related to palliative care needs. Also, most studies have assessed the factors related to physical and psychological burden and overall quality of life impacts of serious illness. Also, the table reveals sparse evidence on factors associated with or related to palliative care needs in the information sub-domain (psychological) (*n* = 3), financial sub-domain (social) (*n* = 5) and spiritual domains (*n* = 9). Details of the direction of relation are presented in Supplementary File 1.

**Table 3.** Cross-tabulation matrix of factors associated with palliative care needs.

	Domains of palliative care needs						Overall wellbeing and Quality of Life
	Physical	Psychological		Social		Spiritual	
		Emotional	Information	Relationship and practical support	Financial		
Person (Patient and family)	BMI^105,106^ Gender^34,106–111^ Age^34,108,111,112^	Age^111,113–116^ Gender^107,109,110,114,116–119^ Avoidant coping style^120^		Gender^95,97^ Age^121,122^			Age^111,121,123^ Gender^110^ Caregiver burden: Being a female carer^58^
Health and Illness	Disease markers^106,124,125^ Poor physical function^95,97,109^ Diagnosis (cancer vs non-cancer)^32,126^ Depressive symptoms^42^ Disease duration^111^	Diagnosis^107,125^ Poor physical function^95,97,109,114^ Dermatological and Social image related symptoms (Including Body fat redistribution)^127,128^ Disease progression^100,108,109,129^ Disease markers^113,116,118,124,125^ Perceived stress^42^	Disease progression^89^ Diagnosis^82^	Disease progression^89,97,130^ More chronic symptoms^94^ Poor physical function^97^ Disease marker^118,131^ Diagnosis (HIV vs non-HIV)^125^ Dermatological and Social image related symptoms (Including Body fat redistribution)^128^ Severe depressive symptom^99,132^ Low QOL^132^	Disease progression^33,130^	Disease markers^91,131^ Poor physical function^95,97^ Disease progression^133^ Diagnosis (Ca vs non Ca)^125,134^	Disease Progression^108,135^ Poor physical function^109,111,136^ Higher anxiety^137^ Diagnosis (Ca vs non Ca)^134^ Disease markers^125,138^ Depression^139^
Environment	Alcohol dependence^124^ Experience of internalised stigma^124^ Unemployment status^106,124^ Larger family size^140^ Availability of Social Welfare^34,141^ Low income^140^ Lack of insurance coverage^140^	Alcohol dependence^114,124^ Smoking^129^ Experience of internalised stigma^124^ Educational level^115,116,125,142^ Disclosure of diagnosis^114^ Poor social support^114^ Financial difficulties^100,115,137^ Unemployment^124^ Experience of recent violence^114^ Experience of stigma^120,124,143^ Marital status^115,120^ Availability of social welfare^133^ Religiousity^131^	poor social support^94^	Urban migration^42^ Marital Status^94^ Educational level^94,97,125^ Experience of stigma^94^ Disclosure to friends^99^ Availability of social welfare^133^ Low income^132^	Availability of social welfare^133^	Availability of social welfare^133^	Malnutrition^135^ Low Income^123^ Interpersonal relationships^134^ Level of education^125^ Experience of stigma^143^ Availability of social welfare^144^ Religiousity^131,134^
Treatment	Treatment type^51^ Cost of care^82^ Lack of explanation about the cause of pain^34^ Time under care^98,145,146^ Treatment type^51,98,147^ Place of care (home vs facility)^98^ Receipt of spiritual care^148^	Medication non-adherence^114,149^ Dying in a facility^150^ Support group participation^120^ Patient’s knowledge of diagnosis^101,120^ Length of years on treatment^98,99,108^ Treatment type^98,118,147^ Place of care (home vs facility)^98^ side effects of medications^120^		Treatment type^118,147^ Time on treatment^99^	Treatment type^51,146^	Place of care (home vs facility)^98^ Time under care^98^	Treatment type [TB medication]^91,138^ Cost of care^82^ Medication non-adherence^119^ Previous hospital admission^123^ Place of care (home vs facility)^144^ Caregiver Burden: Dying at home^150^

## Discussion

This review provides strong evidence and conceptual clarity for the palliative care needs of people with serious illness and their family members in Africa. The new framework elaborates the domains of the WHO definition using evidence from Africa and across serious illnesses. Our framework included subthemes of emotional and information needs under psychological domain; and financial, relationships and practical support needs under social domain to reflect the evidence.

The results suggest that financial constraints, poor health literacy, poor communication from healthcare workers and cultural and spiritual worldviews of patients with serious illnesses and their families are major drivers of palliative care needs. This reflects the wider context-defined socioeconomic and cultural realities faced by them. Thus, in other to provide palliative care which meets patients’ and families’ physical, psychological, social and spiritual needs within universal health coverage, it is imperative to assess and address palliative care needs using this framework. Therefore, palliative care delivery in these setting must involve a wider systemic approach which focuses on underlying contextual dynamics driving needs.

A previous review^151^ classified palliative care problems of patients with HIV based on whether they are from low, middle or high income countries. They found no evidence for psychological, wellbeing, spiritual, emotional and information or tangible support domains of palliative care problems in HIV patients in inpatient settings from low-and middle-income countries. This undermines the importance of these non-physical domains in the provision of palliative care that is person-centered and not disease-focused for patients on admission. This has also propagated the overemphasis on indicators related to management of physical symptoms such as morphine access in understanding palliative care needs at a population level.^152^ Our study does not differentiate inpatient and outpatient settings; nevertheless, our findings underscore the necessity of a clear overarching framework to ensure palliative care needs of patients with serious illnesses from low-and middle-income countries are not neglected whether they are in hospital or in the community. Simm’s study^151^ also highlighted information need about support resources available within the community. In addition to this, our study demonstrated information needs arising from low health literacy and the need to know and demystify the cause and course of serious illnesses which often cause psychosocial and spiritual distress for patients and families.

Another study established commonalities in palliative care-related problems across selected serious illnesses.^153^ Their findings also highlighted the need for intensifying the measurement and reporting of spiritual and social palliative care–related problems. This agrees with the finding of our study that reporting of physical and psychological needs was higher than other domains (Figure 3). This highlights gaps in evidence of needs in social and spiritual domains. Within the context of low and middle income countries, socioeconomic, cultural and religious factors often interact with the experience of illness to influence palliative care needs in these domains.^154–156^ The non-medical nature of social and spiritual needs mean the meeting of these needs are ignored or assigned to already overburdened families within resource-poor healthcare settings with inadequate social welfare protection. Nevertheless, to provide quality palliative care within universal healthcare coverage, essential package recommendations must not give less importance to these domains.

Furthermore, palliative care needs must be understood within the unique local cultural contexts to improve palliative care delivery for the patients and families across Africa. This is because Africa is a vast continent with extensive cultural diversity. Thus, palliative care providers must be culturally competent to consider the role of the unique worldview of patients and families in the understanding of their illness, their palliative care needs, how these are being communicated and how to meet needs in a person-centred manner.

Our review identified gaps in evidence of the palliative care needs in other serious illnesses apart from HIV/AIDS and cancer (Figure 3). Some previous reviews of palliative care needs have focused only on cancer patients or HIV patients as against the range of illnesses covered in our study.^151,157,158^ Although palliative care advanced in Africa in the context of the HIV/AIDS epidemic and pain management in cancer patients,^159^ recent projections of global mortality and global palliative care needs by 2060^160^ have shown that non-communicable diseases such as cancer, lung diseases, cerebrovascular disease, liver diseases, dementia and non-ischaemic heart diseases will drive the need for palliative care. This changing and emerging trend warrant a broader understanding of palliative care problems and needs in other serious illnesses in this context as espoused in this framework. Nevertheless, outside of HIV and cancer, referrals to palliative care continues to be very low in Africa.^161^

Tools for identifying patients with palliative care needs were developed in high-income countries and shown to be largely inaccurate.^162^ In many African cultures, there is also an inherent aversion for prognosticating^163,164^ on which many of these tools are based on. This means many patients who need palliative care are not identified on time and live with unnecessary suffering. A recent study has proposed the use of tools based on the anticipation of palliative care needs as alternatives for the identification of patients who need palliative care within primary healthcare.^162^ Our finding provides comprehensive evidence for the development of such tools for improving palliative care referrals for patient with serious illnesses in Africa.

Furthermore, palliative care is not only person-centred but also family-focused care.^165^ Family caregivers play essential roles in supporting patients and weak health systems in Africa. To our knowledge, this is the first review to articulate the unique yet often hidden multidimensional palliative care needs of family caregivers of patients with serious illnesses in Africa. We have shown that family caregivers of patients with serious illnesses have physical, psychological, social and spiritual needs as well (Table 2). Yet, these are often ignored as the focus rest on the patients. For instance, in defining palliative care problems, the current WHO guide for integrating palliative care and symptom relief into primary healthcare stated that ‘*these problems include physical, psychological, social and spiritual suffering of patients and psychological, social and spiritual suffering of family members*.’^27^ This disregarded physical needs in families as important considerations for palliative care service planners, implementers and managers. Our findings show that physical needs in families must not be neglected as the burden of caregiving roles impact family-caregivers’ health. This is even more critical in low-and-middle income countries where poorly supported and overwhelmed family members lack the time and resources to seek care for their own physical symptoms. This necessitates adequate consideration of the palliative care needs of the family caregivers to ensure their unique needs are addressed. This will also impact on the health and wellbeing of the patient.

### Strengths and limitations

This study reviewed a large body of work providing in-depth understanding of palliative care need in the African context and was conducted by a group of authors with contextual knowledge, using an international framework to guide analysis. Some factors may limit the interpretation of our findings. First, analysing a concept should not be limited to its use in research alone but wider use outside research and practice. Our search strategy was designed to capture all the relevant papers that addressed an area of need relevant to palliative care. Although we conducted a comprehensive and broad search of the literature, some papers may have been missed considering the nature of the concept of need. Second, Health literacy is a critical factor in patients’ understanding of their needs and in building the self-efficacy to seek care that addresses their needs.^166^ Coding the reporting of information needs was challenging as some studies did not necessarily report this domain separately but reported for example that patients believed that the cause of the disease was witchcraft.

Third, for the geographic mapping, some studies span more than one African country with data not usually disaggregated by country. To deal with this in the mapping of the studies, the studies were counted as one for each of the countries in which the study was conducted. Fourth, our review focused on adult patients and our Africa-specific framework of palliative care needs may not apply in paediatric patients. We also excluded children who are family caregivers of adult patients with serious illness from this review. The burden of caregiving sometimes falls on young children in Africa^167,168^ as stigma leads to declining social networks especially in the context of HIV/AIDS.

## Conclusions

Our review presents strong evidence and conceptual clarity for the specific palliative care needs of people with serious illness and their families in Africa. The comprehensive overarching framework developed elaborates on the domains of palliative care needs highlighted in the WHO definition using evidence from Africa and across different serious illnesses. We identified critical gaps in evidence of palliative care needs in west, central and North Africa, in serious illnesses other than HIV/AIDS and Cancer and in family caregivers.

Thus, to achieve the outcomes improvement and cost-saving effects of palliative care within Universal Health Coverage in Africa, it is imperative to address total care needs in line with the African evidence-based framework identified in this review. Based on contextual factors driving palliative care needs, health literacy is a specific and crucial need that must be met to ensure that the benefits of palliative care can be achieved at the patient-level. The WHO must review and update the recommendations for planners and implementers of palliative care within primary healthcare to ensure families have access to adequate support for their palliative care needs. The framework should also inform clinical multidimensional needs assessment of patients with serious illnesses in Africa in a way that encompasses context-driven needs. The recommendations in the essential package for palliative care within universal health coverage must be updated to put equal emphasis on the social and spiritual needs as physical and psychological needs in this context. Therefore, there is need to develop and test palliative care service models which build on this framework to broaden comprehensive palliative care delivery across serious illnesses in Africa.

## Supplemental Material

sj-pdf-1-pmj-10.1177_02692163211008784 – Supplemental material for What constitutes a palliative care need in people with serious illnesses across Africa? A mixed-methods systematic review of the concept and evidence Click here for additional data file. Supplemental material, sj-pdf-1-pmj-10.1177_02692163211008784 for What constitutes a palliative care need in people with serious illnesses across Africa? A mixed-methods systematic review of the concept and evidence by Oladayo A Afolabi, Kennedy Nkhoma, Matthew Maddocks and Richard Harding in Palliative Medicine

sj-pdf-2-pmj-10.1177_02692163211008784 – Supplemental material for What constitutes a palliative care need in people with serious illnesses across Africa? A mixed-methods systematic review of the concept and evidence Click here for additional data file. Supplemental material, sj-pdf-2-pmj-10.1177_02692163211008784 for What constitutes a palliative care need in people with serious illnesses across Africa? A mixed-methods systematic review of the concept and evidence by Oladayo A Afolabi, Kennedy Nkhoma, Matthew Maddocks and Richard Harding in Palliative Medicine
