# Supplementary material for: What constitutes a palliative care need in people with serious illnesses across Africa? A mixed-methods systematic review of the concept and evidence
Source: Palliat Med. 2021 Apr 16;35(6):1052–70. doi: 10.1177/02692163211008784 (PMC8371282; doi:10.1177/02692163211008784)
Supplement: sj-pdf-1-pmj-10.1177_02692163211008784 – Supplemental material for What constitutes a palliative care need in people with serious illnesses across Africa? A mixed-methods systematic review of the concept and evidence [file sj-pdf-1-pmj-10.1177_02692163211008784.pdf]

SUPPLEMENTARY FILE 1: Full data Extraction

S/No	Author	Year	Country	Study design<br>Study aim / question	Diagnosis and stage	Domains of Palliative care needs						Coded Themes from Qualitative studies	Factors Associated with palliative care needs	MMAT * Quality Appraisal
						Physical	Emotional	Relationship and Practical support	Spiritual	financial	Information			
1.	Ratshikana-Moloko et al	2020	South Africa	Quantitative<br>To identify religious/ spiritual needs among patients with advanced cancer receiving palliative care services and to assess associations of receipt of R/S care with patient QoL and place of death	Advanced cancer	Yes	Yes	Yes	Yes	No	No		Receipt of spiritual care was associated with reduced pain and family worry (OR 0.33; 95% CI 0.11-0.95; P=0.04 and OR 3.43; 95% CI 1.10-10.70; P=0.03, respectively).	6
2.	Mkandawire-Valhmu et al	2020	Malawi	Qualitative<br>To describe the lived experience of female palliative care patients in rural Malawi and their caregivers.	Advanced cancer	Yes	Yes	Yes	No	Yes	No	Caregiving is isolating and overwhelming<br>Taking time off school<br>Gendered impact on women<br>Increasing poverty over the course of illness<br>Exacerbated domestic violence against women<br>Abandonment and family breakups over patient's inability to provide sexual satisfaction<br>Physical and emotional drain from sexual coercion<br>Experience of stigma<br>Feeling like a burden<br>Expressing feelings of sadness		6
3.	Kusi et al	2020	Ghana	Qualitative<br>To explore and describe the caregiving motivations and experiences among family caregivers of patients living with advanced breast cancer	Advanced cancer	Yes	Yes	Yes	Yes	Yes	No	Finding meaning in caregiving (Caregiving as an obligation and a repayment opportunity)<br>Supplementing cost of care<br>Need for training in medication management (changing dosage and		5

												schedule of medications without prescription)		
**4.**	Kizza et al	2020	Uganda	Quantitative<br>To explore the determinants of Quality of Life among Family Caregivers (FCG) of Advanced Cancer Patients (ACP) in Uganda	Advanced cancer	Yes	No	Yes	No	Yes	Yes		The FCGs perceived burden was significantly associated with education level (p =0.000), perceived impact of caregiving on physical health (p= 0.000), self-rated health (p= 0.008), self-efficacy for cancer pain management (p= 0.003), knowledge about cancer pain management (p= 0.009), ACP's functional status (p= 0.001), levels of ACP pain (p=0.000) and duration of ACP pain (p=0.014).<br>The level of Positive Adaptation and Financial Concerns (PAFC) was significantly associated with the FCGs' level of education (p= 0.012), perceived impact of caregiving on physical health (p = 0.000), knowledge about cancer pain management<br>(p = 0.028), self-efficacy for cancer pain management (p= 0.003) and pain levels in ACPs (p= 0.008).	5
**5.**	Muliira et al	2019	Uganda	Quantitative<br>To explore the tasks performed and the caregiver burden experienced by	Advanced cancer	No	Yes	Yes	No	No	No		The main predictors of overall FCGs' perceived caregiver burden were	5

				FCGs of hospitalized ACPs in a sub-Saharan country									the level of education (P= 0.018), length of stay in the hospital (P =0.031), and performing the task of giving medications to the ACPs (P= 0.049)	
**6.**	Mullira et al	2019	Uganda	Quantitative<br>To describes the extent of depression and anxiety<br>symptoms among FCGs of ACPs and the associated modifiable factors.	Advanced cancer	Yes	Yes	No	No	No	No		The significant predictors of clinical significant depressive symptoms were; time since confirmation of ACP cancer diagnosis (OR=0.49, CI=0.29–0.85), ACP's level of pain (OR=1.34, CI=1.03–1.75), ACP's functional status (OR=0.61, CI=0.44–0.85), self-rated health status (OR=2.24, CI=1.25–4.00) and perceived impact of caregiving on physical health (OR=2.18, CI=1.43–3.323).<br>FCG self-rated health (OR=2.01, CI=1.161–3.488) and perceived impact of caregiving on their physical health (OR=2.04, CI 1.372–3.033), significantly predicted anxiety symptoms in FCGs.	5
**7.**	Ahlam et al	2019	Morocco	Quantitative<br>To evaluate the quality of life of Moroccan patients with advanced palliative cancer.	Palliative phase (not receiving chemotherapy or	Yes	Yes	No	No	No	No		Being a woman predicts report of dyspnoea<br>Being age 30yrs and under predicted poor QOL	5

					radiotherapy) of cancer								Age is associated with emotional functioning<br>Being greater than 70yrs old is associated with pain<br>Duration of disease associated with severity of nausea and vomiting and loss of appetite<br>physical function, emotional function and fatigue were predictors of quality of life	
**8.**	Agom et al	2019	Nigeria	Qualitative<br>To explore ways in which cancer patients, their families and healthcare professionals construct the meaning of their illness and how this impacon the provision of palliative care	Life limiting illnesses	No	No	No	Yes	No	Yes	Belief in spiritual and witchcraft causation of life limiting illness<br>Cultural interpretation of physical suffering<br>False hopes on cure<br>Suppressing awareness of impending death<br>Believing God for cure<br>Rejecting medical advice		6
**9.**	Agbokey et al	2019	Ghana	Qualitative<br>To explore the health seeking behaviour of BC patients and their knowledge of BC in a breast cancer management Centre of Komfo Anokye Teaching Hospital in Ghana.	Breast Cancer	Yes	Yes	No	Yes	Yes	Yes	Perception of BC as a punishment or witchcraft<br>Ignorance and confusion about the disease causation<br>Physical symptoms such as nipple ache, pain, nausea and itchiness<br>Unaffordable medication<br>Anxiety about the procedures<br>Praying and hoping for strength to endure pain		4
**10.**	Bonsu et al[26]	2019	Ghana	Qualitative<br>To explore the reasons for delayed presentation in Ghanaian women with breast cancer.	Advanced breast cancer	Yes	Yes	Yes	Yes	No	Yes	Alternative healing systems/ Pluralism in health<br>Debilitating nature of symptom<br>Attributing cause to evil spirit<br>Lack of knowledge about cancer and its outcome<br>Cancer as God's punishment		6

**11.**	Chang et al [148]	2018	Uganda	Quantitative<br>To estimate associations between efavirenz use and depression and suicidal ideation among PLHIV in Uganda.	HIV	No	Yes	No	No	No	No		ART (efavirenz) has 40% odds of decreasing depression	6
**12.**	Fink et al[95]	2018	Nigeria	Quantitative<br>To measure the prevalence of MSK symptoms in PLWH in urban West Africa.	HIV	Yes	No	No	No	No	No		Higher BMI significantly associated with pain in HIV (p=0.01)<br>Individuals with chronic pain reported stopping work due to musculoskeletal pain (6/28, 21%)	4
**13.**	Gwyther et al [27]	2018	south Africa	Quantitative<br>to describe the access of patients with advanced chronic illness to PC services.	Advanced cancer<br>HIV with CD4 count of <200 cells/mm3<br>Motor neuron disease (MND)	Yes	Yes	No	Yes	No	Yes		Social needs and information domains worsened overtime in HIV. Only physical symptoms improved for cancer<br>The most significant improvement in HIV were pain and worry	5
**14.**	Hamdi et al [149]	2018	Senegal	Quantitative<br>To assess the capacity and need for palliative care in Senegal	Cancer<br>Stroke<br>Chronic heart failure<br>HIV/AIDS	Yes	Can't tell	No	No	No	No		Health providers knowledge of and experience prescribing morphine was limited social support enhanced treatment adherence	4
**15.**	Mugusi et al [118]	2018	Tanzania	Quantitative<br>Incidence of neuropsychiatric manifestations during early initiation of efavirenz-based cART	HIV with comorbid TB	Yes	Yes	No	No	No	No		More patients with neuropsychiatric manifestations being in WHO stages III and IV significantly more psychological problems in smokers (p=0.03)<br>No significant differences were seen in median efavirenz concentrations among	5

													patients with and without neuropsychiatric manifestations at 4 weeks	
16.	Ndetei et al [119]	2018	Kenya	Quantitative examined the effect of a cancer diagnosis on psychological well-being and social functioning across cancer stages.	Cancer	No	Yes	Yes	No	No	No		Suicide were mostly between 25 and 46 years of age. With progression of cancer, there is increasing inability to work, number of days bedridden, and health-related problems causing difficulty with getting along with people in the last 30 days.	5
17.	Ndiok et al [28]	2018	Nigeria	Quantitative to assess the care needs of oncology in-patients and clinic attendees or families in two tertiary health institutions.	Cancer	Yes	Yes	Yes	Yes	Yes	Yes			4
18.	Nkhoma et al [29]	2018	Kenya	Quantitative to measure problems and concerns among HIV patients attending outpatient clinic.	HIV with comorbid TB	Yes	Yes	Yes	Yes	No	Yes		TB treatment was associated with worse symptoms and concerns higher CD4 count was predictive of lower (worse) scores for factor 3 (existential and spiritual wellbeing	6
19.	O'Neil et al [138]	2018	South Africa	Quantitative To better understand the challenges of informal caregivers at the end of life in South Africa, both at home and in inpatient facilities	Advanced Cancer HIV comorbidity 21.8%	Yes	Yes	Yes	Yes	No	No		Overall burden of physical and emotional symptoms reported by patients on their last APCA POS before death showed no association with extent of caregiver difficulty. Caregivers of patients who died at home reported greater difficulty managing pain,	5

													insomnia and fatigue, and when "interacting with the patient" Caregivers of patients dying in a facility reported greater difficulty with shame and sadness	
**20.**	Shearer et al [102]	2018	South Africa	Quantitative to describe the prevalence of depressive symptoms and outcomes one year after screening among patients receiving ART at a large HIV Clinic in Johannesburg, South Africa.	HIV	No	Yes	No	No	No	No		Patients under age of 30 years were more likely to report depression compared to those older than 30 years Those with lower CD4 count <200cells/mm3 and higher viral load >1000copies/mL at ART initiation were more likely t report depression No influence of employment status on report of depression. Nevertheless, depression was only observed in 7% of the population	5
**21.**	Shen, et al [30]	2018	South Africa	Quantitative examined patients' terminal illness awareness, their preferences for the type of care received at EoL, and their current and preferred communication surrounding poor prognosis	Advanced cancer	No	No	No	No	No	Yes			4
**22.**	Bates et al [31]	2018	Malawi	Qualitative To explore concepts of wellbeing and the contribution of palliative care to wellbeing from the perspective of patients and families affected by advanced cancer	Advanced cancer	Yes	Yes	Yes	Yes	Yes	Yes	Transportation help What is a good day? Interpreted as quality of life Lack of appropriate messages available about signs and symptoms Thinking they have been bewitched		6
**23.**	Jones et al [32]	2018	Uganda	Qualitative to evaluate the lived experience of people with CRD, including physical	Post TB Lung	Yes	Yes	Yes	No	Yes	Yes	Economic experiences		5

				and psychosocial impacts, and how these are addressed by PR	disease COPD							Regaining manhood and ability to fulfil conjugal duties might be considered improved quality of life		
**24.**	Kimani et al [33]	2018	Kenya	Qualitative<br>to explore the experiences of patients living and dying with heart failure in Kenya.	Heart failure NYHA Stage III & IV	Yes	Yes	Yes	Yes	Yes	Yes	Financial issues<br>Acceptance, helplessness and praying<br>Cost of medications balanced with need to pay school fee for children		5
**25.**	Copeland [34]	2018	Kenya	Mixed<br>uses a cognitive anthropological approach that conceives of culture as shared models and explores the relationship between how well women know a cultural model of self-managing HIV/AIDS and health among women who are not receiving biomedical treatment.	HIV/AIDS	Yes	Yes	Yes	Yes	Yes	Yes	Good nutrition<br>Reproduction support (pregnancy and breastfeeding)<br>Money<br>care of children	Urban migration increased social isolation<br>Perceived stress positively correlates with depressive symptoms<br>Depressive symptoms positively correlate with reported physical illness	7 of 13
**26.**	Reid et al [35]	2018	Ethiopia	Mixed<br>to assess the overall burden of life-limiting illness, the costs associated with life-limiting illness, and barriers to accessing palliative care in Ethiopia.	HIV<br>Cancer<br>Unspecified NCDs	Yes	Yes	Yes	No	Yes	Yes	Financial hardship<br>Practical support needs for carers<br>Carers have to take time off work or school to care for sick relatives<br>Patients are worried about cost of medical care and have sold assets to offset this<br>Clarity of information poor in hospice and cancer patients	Statistically significant relationship was found between cost of care and pain and cost and wellbeing.<br>HIV patients expressed more clarity around received information than oncology and hospice patient	6 of 13
**27.**	Abdelshafy et al [150]	2017	Egypt	Quantitative<br>evaluated the efficacy, feasibility, and outcomes of SEMS in palliation of malignant dysphagia in advanced cancer esophagus and its' complications	advanced cancer esophagus	Yes	No	No	No	No	No			1
**28.**	Fatiregun et al [126]	2017	Nigeria	Quantitative<br>to evaluate whether significant association exists between anxiety disorders [151] and HRQoL in breast cancer,	Cancer	Yes	No	No	No	Yes	No		Those with higher anxiety disorders have significantly higher financial difficulties and higher symptoms score Those with AD had	6

													statistically significant lower quality of life	
**29.**	Tannor et al [36]	2017	South Africa	Mixed<br>to study the quality of life of patients treated with PD and HD using a comparative mixed methods approach.	Renal Failure	Yes	Yes	Yes	No	No	Yes	Ignorance about kidney disease and inadequate education on organ transplantation	Type of dialysis affects symptoms, and work status. PD patients scored lower with symptoms and sleep, and scored higher for work status and dialysis staff encouragement than those on HD.	10 of 13
**30.**	Wakeham et al [152]	2017	Uganda	Quantitative<br>to assess the impact on symptom burden for the year after ART initiation in individuals with a CD4 count <200 cells/uL in Uganda.	HIV/AIDS	Yes	Yes	No	No	No	No		The prevalence of pain, weight loss, lack of appetite, feeling sad, difficulty sleeping and walking, problems urinating, irritability, feeling nervous and mouth sores reduced in frequency during the early phases of taking ART<br>GDI, PSYCH and PHYS all halved after ART start	5
**31.**	Bates et al [37]	2017	Malawi	Qualitative<br>to describe the palliative care needs of patients with ESKD who were not receiving RRT, at a government teaching hospital in Blantyre, Malawi.,	End Stage kidney disease<br>Estimated glomerular filtration rate < 15 ml/min on two separate occasions, three months apart,<br>Chose not	Yes	Yes	Yes	Yes	Yes	Yes	Financial challenges impacting hospital care<br>Belief in witchcraft is both spiritual and information needs		6

					to have or were not deemed suitable for RRT								
**32.**	Githaiga [94]	2017	Kenya	Qualitative<br>to explore caregiving experiences of women in the context of cancer	Cancer	Yes	No	Yes	Can't tell	No	No	Culturally appropriate practical help for caregivers<br>Vicarious humiliation and shame from providing intimate care for parents<br>Cultural beliefs usually hold some spiritual implication but spiritual needs were not specifically mentioned		5
**33.**	Githaiga [38]	2017	Kenya	Qualitative<br>to explore caregiving experiences of women in the context of cancer	Cancer	No	Yes	No	Yes	Yes	Yes	Inability to afford heavily subsidised care<br>Seeking to make sense of the disease indicate spiritual and information needs<br>Belief in witchcraft<br>Praying for healing<br>Anger from lack of understanding		5
**34.**	Kebede et al [39]	2017	Ethiopia	Qualitative<br>to explore the psychosocial experiences and the needs of women diagnosed with cervical cancer	Cervical cancer	Yes	Yes	Yes	Yes	Yes	Yes	Financial problems<br>Associating cancer as a punishment for sins<br>Deteriorating social network<br>Selling assets and borrowing money to pay for treatment		5
**35.**	Lofandjola et al [40]	2017	Congo DRC	Qualitative<br>to illustrate, in a Congolese context, the perceptions of families on the care of patients suffering from advanced illness, and to identify the possible aids provided by healthcare facilities	not specified	Yes	Yes	Yes	Yes	Yes	Yes	Cost of medications and healthcare<br>Lack of communication between care providers, patients and family<br>Psychological support is lacking<br>Vicarious suffering<br>Helplessness and resorting to prayers		5
**36.**	Namukwaya e al. [41]	2017	Uganda	Qualitative<br>to describe patients' experiences of their illness, their perspectives of their multidimensional needs over the	Heart failure	Yes	Yes	Yes	Yes	Yes	Yes	financial needs<br>Better nutritional support<br>Symptoms only considered significant if they impeded ability to work		6

				illness course and what they and their HPs want to be improved.								Classified financial needs under social needs		
**37.**	Namukwaya et al [42]	2017	Uganda	Qualitative<br>to explore the beliefs of patients with heart failure, their understanding of their illness and its treatment, and how this influenced their health related behaviour to inform future health education programs, information and palliative care services.	Heart failure	Yes	No	No	Yes	No	Yes	Information needs<br>Poor health literacy<br>Lack of awareness of symptoms<br>Dichotomy in illness understanding between patients and professionals<br>No information on self-care		5
**38.**	Githaiga et al [43]	2017	kenya	Qualitative<br>examining the content and contexts of family end-of-life conversations and decisions based on the retrospective accounts of a sample of bereaved women family cancer caregivers in Nairobi.	unspecified terminal illness	No	No	Yes	No	No	Yes	Information and social needs around initiating and managing end of life discussions		5
**39.**	Ohemeng et al [92]	2017	Ghana	Qualitative<br>examines the views of persons living with AIDS about how they want to die and how they are planning for their deaths.	HIV/AIDS	No	Yes	No	Yes	Yes	No	Becoming a financial burden to relatives<br>Psychological- worried about prolonged period of illness and emaciation<br>Spiritual meanings of desire to die after 3 days		4
**40.**	Oyegbile et al [44]	2017	Nigeria	Qualitative<br>To describe the experiences of family caregivers providing care for patients living with End-Stage Renal Disease in Nigeria	End stage renal disease	Yes	Yes	Yes	Yes	No	Yes	Needing a break<br>Relentless caregiving imposed restrictions on caregiver's lives<br>Participants developed severe back pain and unable to cope with their own personal symptoms<br>Needing a break- need for practical support<br>Unending anticipatory grieving poses psychological issues<br>lack of information makes caregivers feel they are being treated as foolish<br>Dealing with spiritual implications of cultural taboos		5

**41.**	Oyegbile et al [45]	2017	Nigeria	Mixed<br>To explore the caregiver burden of family caregivers of End-Stage Renal Disease (ESRD) patients in South-West Nigeria.	End stage Renal disease	Yes	Yes	Yes	No	Yes	Yes	Practical need for carers<br>financial need<br>Majority of carers felt their health has suffered because of caring duties<br>Majority of carers felt they do not have enough finances to care for the sick relative<br>Carers felt uncertain about what to do about the patient's illness	Female caregivers had greater mean caregiving burden than males but it was not statistically significant	10 of 13
**42.**	Edwin et al [153]	2016	Ghana	To determine whether a structured approach to end-of-life decision-making directed by a compassionate interdisciplinary team would improve the quality of care for patients with terminal illness in a teaching hospital in Ghana.,	Not specified	Yes	Yes	Yes	No	No	No			1
**43.**	Harding et al [110]	2016	south Africa	Quantitative<br>(1) identify most burdensome problems,<br>(2) compare intensity of problems for drug-susceptible and drug-resistant tuberculosis<br>(3) identify predictors of problem identifiers.	Tuberculosis<br>Multidrug resistant Tuberculosis	Yes	Yes	Yes	Yes	No	No		Age was predictive of a higher (worse) score for total POS score and Factor 2 (interpersonal wellbeing).	6
**44.**	Lazenby et al [97]	2016	Botswana	Quantitative<br>to describe symptom burden and functional dependencies of cancer patients in Botswana using the Memorial Symptom Assessment Scale-Short Form (MSAS-SF) and Enforced Social Dependency Scale (ESDS).	Cancer Comorbidity with HIV	Yes	Yes	Yes	No	No	No		More HIV+ patients reported feeling sad<br>Being a woman was significantly associated with lack of energy and worrying symptoms	5
**45.**	Lokker et al. [112]	2016	South Africa	Quantitative<br>to measure patient-reported symptom prevalence and correlates of symptom burden in patients with advanced heart failure.	Advanced Heart Failure	Yes	Yes	No	No	Can't tell	No		Age, income and previous hospital admission were correlated with GDI, Physical symptoms and number of symptoms<br>Previous hospital	5

													admission was correlated with psychological distress and total distress Higher symptom burden was associated with older age, having no income and fewer hospital admissions	
**46.**	Machuki et al [154]	2016	Kenya	Quantitative describes quality of life in patients with gynaecological cancer attending Kenyatta National Hospital, Kenya.	Gynaecological cancers	Yes	Yes	Yes	Yes	No	No			4
**47.**	Tesfaye et al [124]	2016	Ethiopia	Quantitative to investigate whether food insecurity and CMDs are associated with lowered quality of life in PLHIV,	HIV	No	Yes	No	No	No	No		Increasing severity of food insecurity was associated with lower quality of life and CMD symptoms Having advanced HIV disease (β = -3.80, 95 % CI: -6.18; -1.42), and having mild malnutrition (BMI = 17.0–18.5 Kg/m2) were also associated with lower quality of life scores (β = -3.45, 95 % CI: -6.18; -0.71)	5
**48.**	Wouters et al [131]	2016	South Africa	Quantitative to investigate the impact of a wide range of individual-level, family-level and community-level determinants of depression	HIV/AIDS	No	Yes	Yes	No	No	No		Respondents' educational level was significantly and negatively correlated with depression with lower educated displaying higher depression Attachment was significantly and negatively correlated with the HADS-D factor: patients residing in a	4

													close-knit family report lower levels of depressive symptoms	
49.	Combrink et al. [46]	2016	South Africa	Qualitative to study the experiences of patients and their families when transitioning from palliative care with anticancer treatment to palliative care without anticancer treatment.	Cancer	Yes	Yes	Yes	Yes	No	Yes	Family: lack of practical assistance in caring for patient Unrealistic expectations mean information need Family did not feel prepared for the impending death Family members not engaged in decision making Family don't know how to care for patient by themselves		5
50.	Mkwinda et al [47]	2016	Malawi	Qualitative explored the needs of PLWHA concerning care from primary caregivers and palliative care nurses in palliative care in Malawi.	HIV/AIDS	Yes	No	Yes	Yes	Yes	Yes	Need for financial assistance for medications, transportation and Children's school fees Need for good nutrition		4
51.	Bates et al [48]	2015	Malawi	Quantitative To describe the symptom burden, palliative care interventions, and outcomes of cervical cancer patients who entered care at Tiyanjane Clinic in Blantyre, Malawi, between January and December 2012.,	Cervical Cancer	Yes	No	No	Yes	No	Yes			3
52.	Lifson et al [49]	2015	Ethiopia	Quantitative to quantify levels of perceived social support and factors associated with low support levels in this population.	HIV	Yes	No	Yes	No	No	Yes		Lower HIV knowledge score is significantly associated with lower social support scores. Lower social support significantly associated with lower education, being widowed, divorced or separated, more chronic symptoms and stigma experiences	3
53.	Maluccio et al [130]	2015	Uganda	Quantitative examined the impact of a food assistance intervention on HRQoL of PLHIV.	HIV	Yes	Yes	No	No	Yes	No		Food assistance statistically significantly increased physical health score PHS of the	5

													MOSHIV but no significant effect on MHS<br>Food assistance decreased the number of reported physical symptoms	
54.	Moens et al [116]	2015	South Africa Uganda	Quantitative<br>to identify and compare symptom clusters among people living with HIV attending five palliative care facilities in two sub-Saharan African countries.	HIV	Yes	Yes	No	No	No	No		Psychological burden was greatest in patients with dermatological-related symptom clusters and social and image related cluster	4
55.	Namisango et al [155]	2015	Uganda	Quantitative<br>to: determine clusters of patients with similar symptom combinations; describe symptom combinations distinguishing the clusters; and evaluate the clusters regarding patient socio-demographic, disease and treatment characteristics, quality of life (QOL) and functional performance.	HIV	Yes	Yes	No	No	No	No			4
56.	Shimakawa et al [156]	2015	Gambia	Quantitative<br>to determine the symptom prevalence and burden among patients with HCC in The Gambia.	Chronic Liver diseases	Yes	Yes	Can't tell	Can't tell	No	Can't tell			4
57.	Andersen et al [88]	2015	South Africa	Qualitative<br>to describe the experience of depression among a sample of peri-urban Black South Africans living with HIV.	HIV/AIDS	Yes	Yes	No	No	No	No	Hopelessness<br>Somatising psychological problems (fatigue, sleep problems)		3
58.	Maree et al [50]	2015	South Africa	Qualitative<br>to elicit the experiences of underprivileged women being confronted with cervical cancer.	Cervical cancer	Yes	Yes	Yes	No	No	Yes	Things left unsaid in breaking news of diagnosis continue to reinforce misunderstanding and worry about illness<br>Support from family member when news was being relayed		4

**59.**	Mkandawire et a [91]	2015	Malawi	Qualitative<br>to examine the links between housing and health among people living with HIV/AIDS (PLWAs) in Northern Malawi	HIV/AIDS	No	Yes	Yes	No	Yes	No	Housing to enable home care<br>Housing arrangements inconvenient for home-based care<br>Highlighted wellbeing in topic but not apparent in results.<br>Lack of resources makes following information given difficult.<br>Lacking peace as a result of worrying about food<br>Cost of house rents compete with nutritional needs for meagre resources<br>Cultural property rights may add to the burden of bereaved families		5
**60.**	Mkwanazi et al [51]	2015	South Africa	Qualitative<br>to better understand women's experiences of living with HIV over a long period of time, and to explore their experiences of participating in the VTS and Amagugu interventions	HIV/AIDS	No	Yes	Yes	No	No	Yes	Perplexed about connection between CD4 count and physical health		5
**61.**	Mkwinda et al [52]	2015	Malawi	Qualitative<br>explored the primary caregiver's needs concerning care given to HIV/AIDS patients and the support they receive from palliative care nurses in Malawi	HIV/AIDS	No	No	Yes	No	Yes	Yes	Financial resources<br>Clinical supplies<br>Respite needs		4
**62.**	Too et al [90]	2015	Uganda	Qualitative<br>to understand what motivated patients and their families to seek formal healthcare, whether there were any barriers to help- seeking and how the help and support provided to them by HAU was perceived.	HIV/AIDS	Yes	Yes	Yes	Can't tell	No	No	Mentioned going to witchdoctor for cure but not specific on information and spiritual needs<br>Poverty as both a barrier and facilitator to health-seeking		4
**63.**	Farrant et al [98]	2014	South Africa	Quantitative<br>To measure the seven-day period prevalence, burden and correlates of pain and other physical and psychological symptoms among HIV patients receiving antiretroviral therapy (ART).	HIV	Yes	Yes	No	No	No	No		Later disease stage and length of years on Rx was associated with worse psychological symptom burden, global symptom burden and number of symptoms	4

													Older age and female gender associated with higher physical symptoms	
**64.**	Harding et al [53]	2014	Uganda Kenya	Quantitative<br>to measure the three-day period intensity of multidimensional problems (physical, psychological, social, and spiritual) among advanced cancer patients in Kenya and Uganda	Malignancy not responsive to curative treatment	Yes	Yes	Yes	Yes	No	Yes		Physical and psychological wellbeing and Existential and spiritual wellbeing improved with older age and worsened with poor physical function<br>Interpersonal wellbeing improved with being male	5
**65.**	Harding et al [125]	2014	South Africa, Uganda Kenya	Quantitative<br>to measure multidimensional wellbeing among advanced HIV and/or cancer patients in three African countries, and determine the relationship between two validated outcome measures.	Advanced HIV and / or Cancer	Yes	Yes	Yes	Can't tell	No	Can't tell		Worsening functional status significantly predicts worse outcome on FACITG+PAL and APOS<br>Palliative care outcomes not significantly different based on Gender and diagnosis	5
**66.**	Hartwig et al [157]	2014	Tanzania	Quantitative<br>To demonstrate the effectiveness of palliative care teams in reducing patients' pain and in increasing other positive life qualities in the absence of morphine; and to document the psychological burden experienced by their clinical providers, trained in morphine delivery, as they observed their patients suffering and in extreme pain.	Cancer	Yes	Yes	Can't tell	Can't tell	No	Can't tell			5
**67.**	Herce et al. [54]	2014	Malawi	Mixed<br>to evaluate early NPCP outcomes and better understand palliative care needs, knowledge, and preferences.	HIV<br>Cancer<br>Stroke<br>Cirrhosis<br>Peripheral Neuropathy	Yes	Yes	Yes	Yes	Yes	Yes	Socioeconomic<br>Transportation and distance problems<br>Housing issue and not having enough money	Pain severity was significantly higher in cancer patients versus non cancer	8 of 13

					Other (Anal Fissure, Paraplegia, TB)							Concerns about ability to perform adequately as a caregiver with no assistance<br>Concerns about ability to perform adequately as a caregiver indicating need for support and training<br>Helplessness, stress and anger<br>Information needs on illness process, medications and care plan<br>Uncertainty and fear		
**68.**	Namisango et al [99]	2014	Uganda	Quantitative<br>to measure seven-day-period prevalence of symptoms among HIV-infected adult outpatients and determine if self-reported symptom burden is associated with antiretroviral therapy (ART), CD4 T-cell count, and clinical disease stage.	HIV	Yes	Yes	No	No	No	No	Memorial Symptom Assessment Schedule – Short Form (MSAS-SF)<br>Karnofsky Performance Scale (KPS)	Patients with KPS score <70 had more symptoms with higher symptom distress.<br>ART and CD4 count were not associated with symptom burden<br>WHO clinical stage was associated with psychological symptom burden<br>Men more likely to experience higher symptom burden	5
**69.**	Omoyeni et al [2]	2014	Nigeria	Quantitative<br>to review the spectrum of adult cancer patients involved in home-based palliative care, the services provided, outcome and benefits.	Cancer	Yes	Can't tell	Can't tell	No	Yes	Yes			4
**70.**	Seth et al [103]	2014	Kenya<br>Namibia<br>Tanzania	Quantitative<br>describes overall psychosocial functioning and factors associated with depressive symptoms among PLHIV attending HIV care and treatment clinics in Kenya, Namibia, and Tanzania.	HIV	Yes	Yes	Yes	No	No	No		Greater levels of depressive symptoms were associated with: (1) being female, (2) younger age, (3) not being completely adherent to HIV medications, (4) likely dependence on alcohol, (5) disclosure to three or	5

													more people (versus one person), (6) experiences of recent violence, (7) less social support, and (8) poorer physical functioning	
**71.**	Modeste et al [55]	2014	South Africa	Qualitative<br>to explore and describe the perceived sources of information as well as the types of information available with regard to self-care symptom management strategies received by women living with HIV in an urban area in the eThekwini district in KwaZulu-Natal.	HIV	No	No	No	No	No	Yes	Information needs<br>Self-care information needs by try and error<br>Personal network as information source		4
**72.**	Streid et al [56]	2014	Uganda South Africa	Qualitative<br>What are the stressors experienced by caregivers of patients receiving palliative care in South Africa and Uganda. What kinds of resources do these caregivers draw on?	HIV	Yes	Yes	Yes	Yes	Yes		Financial hardship<br>Fatigue and sleeplessness from caring duties<br>Helplessness		5
**73.**	Elumelu-Kupoluyi et al [158]	2013	Nigeria	Quantitative<br>to assess the pain and discomfort of cancer patients with stage II secondary lymphedema and the effectiveness of available treatment options	Cancer	Yes	No	No	No	No	No			5
**74.**	Harding et al [159]	2013	Tanzania	Quantitative<br>to determine whether palliative care delivered from within an existing HIV outpatient setting improves control of pain and symptoms compared to standard care.	HIV	Yes	Yes	Can't tell	Can't tell	No	Can't tell		CD4 count and ARV use were not associated with improved MOS-HIV MHS and PHS scores	6
**75.**	Jaquet et al [108]	2013	Burkina Faso	Quantitative<br>to assess the temporal changes and factors associated with HRQOL among HIV-positive adults initiating HAART in Burkina Faso.	HIV	Yes	Yes	Yes	No	No	No		The use of HAART was associated with a significant increase in both physical and mental aspects of the MOS-SF 36 HRQOL<br>Women had significant	5

													increase in MHS score at 12 months of HAART compared to men. Discrimination history not significantly associated to MHS & PHS scores	
**76.**	Morwe et al [57]	2013	South Africa	Quantitative<br>explored the profile of HIV and AIDS caregivers in Thohoyandou in South Africa.	HIV	No	No	No	No	Yes	Yes			2
**77.**	Nel et al [137]	2013	South Africa	Quantitative<br>to determine the severity of symptoms of depression and anxiety among a South African sample of patients receiving ART in a public HIV clinic.	HIV	No	Yes	No	No	No	No		Patients with poor ART adherence are approximately three times more likely to report moderate to severe symptoms of depression	4
**78.**	Peltzer [113]	2013	South Africa	Quantitative<br>to determine the prevalence, predictors, and self-reported management of HIV- or ARV-related symptoms among HIV patients prior to antiretroviral therapy (ART) and over three time points while receiving ART	HIV	Yes	Can't tell	Yes	No	Yes	No		A higher symptom frequency amongst patients who were not employed, had lower CD4 cell counts, experienced internalised stigma, and used alcohol.	6
**79.**	Shumba et al. [106]	2013	Uganda	Quantitative<br>to describe the prevalence of depressive symptoms among PLHIV in AIDSRelief (AR)	HIV	No	Yes	No	No	No	No		Majority of the patients on highly active antiretroviral therapy (HAART) (59%) were found to have depressive symptoms and this was significantly more among women than men (66% vs 43%)	4
**80.**	Simms et al [58]	2013	Uganda Kenya	Quantitative<br>to determine for the first time the prevalence and severity of multidimensional problems in a population newly diagnosed with HIV at outpatient clinics in Africa.	HIV	Yes	Yes	Yes	Yes	No	Yes		Patients with limited physical function reported significantly more physical/ psychological (OR = 3.22) and existential	6

													problems (OR = 1.54) but fewer interpersonal problems (OR = 0.50). All outcomes were independent of CD4 count or ART eligibility. Women and those with poor education are significantly more likely to have interpersonal problems<br>More recently diagnosed patients are significantly more likely to have interpersonal problems	
**81.**	Gonzaga [93]	2013	Uganda	Qualitative<br>to explore the lived experiences of women diagnosed and living with breast cancer.	Breast cancer	Yes	Yes	Yes	Yes	No	No	Loosing meaning<br>Questioning God		4
**82.**	Jansen et al [84]	2013	South Africa	Qualitative<br>to explore quality of life from the perspective of palliative care patients managed at a palliative care clinic serving a resource-poor community in Tshwane, South Africa.	HIV<br>TB<br>Stroke<br>Cancer	Yes	Yes	Yes	Yes		No	Poverty negatively infuences QOL<br>Lack of food<br>Life was a daily struggle for survival<br>Today we eat, tomorrow we don't		4
**83.**	Selman et al. [83]	2013	Uganda<br>Kenya	Qualitative<br>to describe the problems experienced by people with HIV in Kenya and Uganda and the management of these problems by HIV outpatient services.	HIV/AIDS	Yes	Yes	Yes	Yes	Yes	No	Financial needs discussed as social needs		6
**84.**	Philips et al [59]	2013	Botswana	Mixed<br>to describe the quality of life and the emotional and spiritual well-being of people at the end of life and the sources of distress for their primary caregivers	not specified	Yes	Yes	Can't tell	Yes	Yes	Yes	Practical concerns for caregivers<br>Need for food and groceries<br>Need support with transportation<br>mentioned training needs for caregivers<br>Physical domain includes physical caregiving needs such as support with bathing, dressing and toileting		6 of 12

**85.**	Evans et al [96]	2012	South Africa	Quantitative<br>investigate the effect of HIV- and TB-related PN on the persistence and recurrence of PN following ART initiation.	HIV	Yes	No	No	No	No	No		Peripheral neuropathy is more likely in male patients, unemployed patients, those with lower median Heamoglobin, lower BMI<br>In addition, TB related PN more likely in slightly younger patients, those with lower median CD4 count<br>PN is associated with higher death rates. and patients with PN at ART initiation are at increased risk of mortality	4
**86.**	Farrant et al [160]	2012	south Africa	Quantitative<br>to measure the prevalence and burden of pain and other physical and psychological symptoms among South African HIV-positive patients attending highly active antiretroviral therapy (HAART) clinics	HIV	Yes	Yes	No	No	No	No			4
**87.**	Harding et al [60]	2012	south Africa Uganda	Quantitative<br>to determine the three-day period intensity of problems (physical, psychological, social and spiritual) among HIV patients receiving integrated palliative care in sub-Saharan Africa, and to identify associations with problem severity.	HIV/ AIDS	Yes	Yes	Yes	Yes	No	Yes		Being longer under care, being on ART were independently associated with improved physical and psychological symptoms<br>Being cared for at home was associated with worse physical and psychological symptoms and worse spiritual wellbeing<br>Poor physical function associated with worse spiritual wellbeing being longer under care	4

													was associated with better existential and spiritual wellbeing	
**88.**	Harding et al [100]	2012	South Africa Uganda	Quantitative to measure the seven-day period prevalence and correlates of physical and psychological symptoms, and their associated burden, in HIV-infected individuals attending palliative care centers in sub-Saharan Africa.	HIV	Yes	Yes	No	No	No	No		Being female and having poor physical function are significantly correlated to worse GDI, physical symptoms burden and number of symptoms and only gender is associated with psychological burden. Family household size, ART use and previous diagnosis of AIDS were not associated with worse symptom burden	4
**89.**	Lewington et al [161]	2012	Uganda	Quantitative (1) determine the point prevalence of inpatients with active life-limiting disease and (2) describe multidimensional need for palliative care among these patients.	HIV/AIDS (61%), cancer (18%), heart failure (9%), renal failure (9%), liver failure (2%) and COPD (1%)	Yes	Yes	Yes	Yes	Yes	No			5
**90.**	Pappin et al [109]	2012	South Africa	Quantitative explores correlates of anxiety and depression in patients enrolled in a public sector ART programme in South Africa.	HIV	No	Yes	Yes	No	No	No		Patients experiencing disruptive side effects of medications and those with avoidant coping style reported more anxiety symptoms. Longer length of time of knowing status and experience of stigma increased likelihood of both anxiety and depression	5

													Patients attending support groups had fewer symptoms of depression . Widows have fewer symptoms of depression than single individuals	
**91.**	Peltzer [122]	2012	South Africa	Quantitative<br>to assess the predictors of the receipt of a disability grant (DG) status and the impact of the DG on health outcomes<br>of HIV patients and on antiretroviral therapy (ART)	HIV	Yes	Yes	Yes	Yes	Yes	No		Receipt of grants was associated with Unemployment, higher psychological, social& spiritual QoL, and higher frequency of HIV symptoms	5
**92.**	Peltzer et al [104]	2012	South Africa	Quantitative<br>assessed the prevalence and predictors of psychological distress as a proxy for common mental disorders among tuberculosis (TB) patients in South Africa	TB	No	Yes	No	No	Yes	No		Older age, lower formal education, not being married, separated divorced or single, poverty were associated to psychological distress.	5
**93.**	Wagner et al [107]	2012	Uganda	Quantitative<br>the impact of ART on mental health outcomes among new clinic patients in Uganda who were followed up for the first 12 months of care	HIV/AIDS	Yes	Yes	Can't tell	No	No	No		Elevated depressive symptoms were significantly much higher in females than males. Those on ART had greater internalised stigma and elevated depressive symptoms than non-ART group CD4 cell count was negatively correlated with depression and internalised stigma	4
**94.**	Kuteesa et al [61]	2012	Uganda	Qualitative<br>examines the medical care experiences of older Ugandans living with HIV.	HIV/AIDS	No	Can't tell	Yes	No	Yes	Yes	Financial ability determines access to care<br>Delayed care seeking due to ignorance		5
**95.**	Mabena et al [62]	2012	South Africa	Qualitative<br>To describe psychological understandings of chronic illness	Cervical cancer	No	Yes	Yes	Yes	No	Yes	The spiritual purpose of illness<br>Praying to resolve fear and worry<br>Witchcraft causation schema protects patient psychologically		6

												from self-blame, guilt and isolation<br>Group support inspires hope		
96.	Makhele et al [63]	2012	Botswana	Qualitative<br>to explore and describe the experiences of Batswana families regarding hospice care for patients	HIV/AIDS	No	Can't tell	Yes	No	No	Yes	Cultural barriers to hospice use<br>Hospice use drives stigma<br>Hospital vs hospice care		5
97.	Dekker et al [64]	2012	South Africa	Mixed<br>to examine patient experiences and health care provider attitudes towards chronic pain and palliative care in Eastern Cape Province, South Africa	HIV<br>TB<br>Renal fialure<br>Cancer	Yes	Yes	No	No	No	Yes	Not having received an explanation for the cause of pain also appeared to be related to increased interference with quality of life.	Individuals who were older, female, with no social welfare grant, or with no explanation for the cause of their pain were more likely to report higher Pain ratings in the last month (adjusted $R^2 = 0.267$, P= 0.004)	4 of 13
98.	Alsirafy et al [162]	2011	Egypt	Quantitative<br>to estimate the extent to which Egyptian patients may be undertreated because of this law.	advanced cancer	Yes	No	No	No	No	No			5
99.	Harding et al [163]	2011	South Africa<br>Uganda	Quantitative<br>to determine the symptom prevalence and burden amongst advanced cancer patients in two African countries.	Cancer	Yes	Yes	No	No	No	No			4
100.	Olagunju et al [164]	2011	Nigeria	Quantitative<br>to determine the prevalence of depression in cancer patients.	Cancer	No	Yes	No	No	No	No			5
101.	Olisah et al dd[128]	2011	Nigeria	Quantitative<br>explored the effect of depressive disorder on the quality of life (QOL) of patients with HIV.	HIV	Can't tell	Yes	Can't tell	No	No	No		Depression did not significantly differ based on gender, age, educational level or occupation<br>Quality of life significantly differ based on presence of symptoms of depression	4
102.	Peltzer [120]	2011	South Africa	Quantitative	HIV	No	Yes	Yes	Yes	No	No		Religious attendance, Private religious activity and intrinsic religiousity	4

				assesses the effects of spirituality and religion in health outcomes of patients on ART									decreased significantly across assessment periods<br>Age was not associated with spirituality or religiousity<br>Depressive symptoms and quality of life were inversely associated with religiousity.<br>Higher CD4 counts were positively associated to intrinsic religiousity	
103.	Peltzer et al [121]	2011	South Africa	Quantitative<br>To examine whether internalized AIDS stigma among HIV patients one year after antiretroviral therapy (ART) initiation is associated with sociodemographic characteristics, health status, social support, quality of life (QoL), and ARV adherence	HIV	Yes	Yes	Yes	Yes	No	No		Not having any income, lower CD4 cell counts, severe depression, and low QOL were predictors of internalised stigma	4
104.	Selman et al	2011	South Africa<br>Uganda	Quantitative<br>to describe QOL among patients with incurable, progressive disease receiving palliative care in South Africa and Uganda, to compare QOL in cancer and HIV, to determine how domains of QOL correlate with overall QOL, and compare levels of QOL in this population with those in other studies using the same tool.	HIV 80.7%<br>Cancer 17.9%<br>Other Conditions (MND, SLE, MS, Korsakoff's syndrome)	Yes	Yes	Yes	Yes	No	No		Spirituality, wellbeing and interpersonal relationships correlated most highly with overall quality of life<br><br>Patients with ca had significantly better wellbeing, spirituality and quality of life than HIV patients	5
105.	Tapsfield et al [165]	2011	Malawi	Quantitative<br>Hospital based palliative care in sub-Saharan Africa; A six month review from Malawi	HIV<br>Cancer<br>Others (liver and/or renal failure, heart failure	Yes	Yes	No	No	No	No			4

					and/or cardiomyopathy), Sub arachnoid haemorrhage									
106.	Grant et al [65]	2011	Uganda<br>Kenya<br>Malawi	Qualitative<br>to describe patient, family and local community perspectives on the impact of three community based palliative care interventions in sub-Saharan Africa.	HIV/AIDS<br>TB<br>Breast cancer<br>Kaposi Sarcoma	Yes	Yes	Yes	Yes	Yes	Yes	Transport to hospital<br>Need for food<br>Need for school fees<br>Providing financial support<br>Financial and pratical problems of dealing with fragmented care<br>Morphine as an enabler of peaceful death<br>Training/ information need on practical care for carers<br>Clinical and emotional needs were intricately connected to the need for food, basic shelter, warmth and school fees		8 of 13
107.	Makoae [66]	2011	Lesotho	Qualitative<br>to explore caregivers' experiences with diagnostic procedures and outcomes, prescriptions and treatment outcomes when ARVs were unavailable	HIV/AIDS	Yes	Yes		Yes		Yes	Cost of buying often expensive medications<br>Caregivers helpless and hopeless		4
108.	Mshana et al [67]	2011	Tanzania	Qualitative<br>'We call it the shaking illness': perceptions and experiences of Parkinson's disease in rural northern Tanzania	Parkinson's disease	Yes	Yes	No	Yes	Yes	Yes	Economic loss<br>Psychological humiliation of carers due to financial burden of illness		5
109.	Bowie et al [166]	2010	Malawi	Quantitative<br>Has the introduction of ART changed the clinical needs for HBC?	HIV- All stages	Yes	No	No	No	No	No			4
110.	Alsirafy et al [167]	2010	Egypt	Quantitative<br>the prevalence of symptoms reported by advanced cancer patients during their first visit to a palliative care clinic in Cairo.	Cancer	Yes	Yes	No	No	No	No			3

111.	Elsharkawy et al [168]	2010	Egypt	Quantitative<br>To present our experience in the use of SEMS in palliation of patients with malignant dysphagia and/or ERF	Cancer Malignant oesophageal stricture	Yes	No	No	No	No	No			3
112.	Fox et al [134]	2010	Kenya	Quantitative<br>to assess wellbeing over their first two years on ART	HIV	Yes	No	No	No	No	No		Seven-day recall of any bodily pain, nausea and fatigue decreased over two years on ART	4
113.	Kabore et al [133]	2010	Lesotho South Africa Namibia Botswana	Quantitative<br>to determine the effect of selected nonmedical supportive care services on health outcomes in patients receiving ART.	HIV	Yes	Yes	Yes	No	Yes	No		Need for financial support increased at the same rate as physical care and psychological needs. Participants who reported receiving food sup-port and/or HBC experienced significant improvement in overall HRQOL at 18 months (57.3 versus 56.0; p ¼ 0.010) compared with those not receiving those services	4
114.	Kagee [169]	2010	South Africa	Quantitative<br>examines the extent to which sub-clinical psychological distress among South Africans living with HIV is any different from patients living with other chronic illnesses, namely diabetes and hypertension as measured by the HSCL	HIV	Can't tell	Yes	No	No	No	No			3
115.	Kagee et al [170]	2010	South Africa	Quantitative<br>to systematically document the extent of symptoms of depression and anxiety among a semi-rural sample of patients in South Africa attending public health clinics.	HIV	Yes	Yes	No	No	No	No			5
116.	Nakasujja et al [171]	2010	Uganda	Quantitative<br>(1) to assess depression symptomatology among HIV-positive patients who were about to initiate HAART and HIV-negative	HIV	Can't tell	Yes	No	No	No	No		No association between CD4 increase and decrease in CES-D scores over 6 months	4

				individuals; (2) to determine the association of depression symptomatology and cognitive function among HIV-positive and HIV-negative individuals; and (3) to evaluate changes in depression symptomatology among HIV-positive individuals receiving HAART.									
117.	Rosen et al [135]	2010	South Africa	Quantitative<br>assessed symptom prevalence, general health, ability to perform normal activities, and employment status among adult antiretroviral therapy patients in South Africa over three full years following ART initiation.	HIV	Yes	No	Can't tell	No	No	No		Being on ART for one and half years increased the probability of getting employment by 45% in unemployed patients<br>Probability of reporting did not fall in the first one year on ART but started to fall at two years and fell by 41% over 3 years of ART<br>Fatigue, nausea and skin problems also declined	4
118.	Wakeham et al [172]	2010	Uganda	Quantitative<br>to measure symptom burden prior to antiretroviral therapy (ART) initiation in a population of adults with low CD4 presenting for human immunodeficiency virus (HIV) care and treatment in Uganda	HIV/AIDS	Yes	Yes	No	No	No	No		Mean total number of physical and psychological symptoms- 14	4
119.	Small [86]	2010	Namibia	Qualitative<br>to describe the experiences of patients receiving haemodialysis for chronic renal failure	Chronic Renal Failure	Yes	No	Yes	No	Yes	No	Financial constraints<br>Medication and treatment expenses<br>Transport expenses		5
120.	Patel et al [136]	2009	Zimbabwwe	Quantitative<br>to assess the impact of ART on HIV-positive women's health-related quality of life	HIV	Yes	Yes	Yes	No	No	No		Treated group had highest mean number of symptoms reported at baseline, lowest current mean number of symptoms, higher mean mental health MOS scores, better scores on	5

													psychosocial measures (UCSF CAPS depression scale and SSQ14) and more likely to disclose, experienced lower social stigma status compared to the others Among those on ART, change in CD4 count and treatment duration were significantly corelated at 55%	
121.	Pearson et al [68]	2009	Mozambique	Quantitative<br>examine whether stigma among patients in a large hospital in central Mozambique 1 year after ART initiation is associated with disclosure decisions, social support, and depression	HIV	No	Yes	Yes	No	Yes	Yes		No gender differences in depression, perceived social support and stigma After 1yr of ART, perceived social support decreased, depression scores increased, negative self-image worsened and perceived social and public stigma increased significantly Lower stigma was significantly reduced by higher disclosure to friends.<br>Depression was significantly related to and contributed most to stigma	6
122.	Selman et al [69]	2009	Uganda South Africa	Qualitative<br>To explore the information needs of patients with progressive, life limiting disease and their family caregivers in South Africa and Uganda and to inform clinical practice and policy in this emerging field.	unspecified incurable progressive illness	No	Yes	No	No	Yes	Yes	Financial support and food<br>Unmet information needs lead to worry		5

123.	Emanuel et al [70]	2008	Uganda	Quantitative<br>to gather pilot data on the circumstances of informal caregivers and the desirability of such programs.	AIDS<br>Cancer	No	Yes	Yes	No	Yes	Yes		Most common cause of worry is patient's illness and financial needs	4
124.	Jameson, C. [12]	2008	South Africa	Quantitative<br>To investigate the palliative care needs of patients with stage 3 and 4 HIV infection in Settlers Hospital, Grahamstown.	HIV stage 3 and 4	Yes	Yes	Yes	No	Yes	Yes			4
125.	Ncama et al [173]	2008	South Africa	Quantitative<br>to examine characteristics related to social support and antiretroviral medication adherence.	HIV/AIDS<br>Co-morbid TB 19.5%	Yes	Yes	Yes	No	No	No		Over half of the sample were not employed (n = 86; 58%).<br>Over 20% of the respondents (22.3%; n = 35) indicated that they had nothing to eat for days during the past week (range = 1–7 days).<br>There were no significant differences in quality of life for those who reported high adherence compared to those who reported low adherence.	5
126.	Peltzer et al. [114]	2008	South Africa	Quantitative<br>to assess the health-related quality of life and HIV symptoms of a sample of people living with HIV (PLHIV) in South Africa	HIV	Yes	Yes	Yes	Yes	Yes	No		Those with AIDS diagnosis scored higher in the domains of spiritual/ religion/ personal beliefs, social relationships and psychological wellbeing and lower on the HIV-symptoms index, than those without AIDS diagnoses<br>Patients with higher CD4 cell count values scored higher in the domains: overall QoL,	5

													psychological health, physical health and independence level, and they scored lower on the HIV-symptoms index than those with lower CD4 cell counts.<br>There were no differences regarding all WHOQOL-HIV BREF domains (except for general health perceptions) and the HIV-symptoms index among Persons treated with antiretroviral medication those who were not on ART.<br>Higher educational levels was associated with higher scores for perceived overall QoL, general health perceptions, psychological health, level of independence, social relationships and environment, and lower scores on the HIV-symptoms index.	
**127.**	Peltzer et al [174]	2008	South Africa	Quantitative<br>to assess HIV symptoms and demographic, social and disease variables of people living with HIV in South Africa	HIV	Yes	No	No	No	Yes	No			5
**128.**	Rosen et al [175]	2008	South Africa	Quantitative<br>examined the association of ART with functional impairment, symptom prevalence, and employment during the first 6 months on therapy.	HIV	Yes	Yes	No	No	No	No		Pre-ART subjects were nearly twice as likely as ART subjects to have suffered any impairment	5

													in the previous week (OR 1.97; 95% CI 1.46–2.66).	
129.	Wingood et al [132]	2008	South Africa	Quantitative<br>to investigate the association between HIV stigma and mental health status among black women living with HIV in the Western Cape	HIV/AIDS	Can't tell	Yes	Can't tell	No	No	No		75.8% unemployed<br>Women reporting more HIV stigma experienced significantly higher depressive symptoms and lower quality of life.	4
130.	Uwimana et al [85]	2007	Rwanda	Mixed<br>to investigate met and unmet palliative care needs for people living with HIV/AIDS in selected areas in Rwanda.	HIV/AIDS	Yes	Yes	No	Yes	Yes	No	Financial needs<br>Nutritional support<br>Housing<br>Spiritual needs was mentioned by only 6% of 250.<br>Psychological need for family carer by only 4%		4
131.	Collins et al [127]	2007	Tanzania	Quantitative<br>measured presenting problems for all patients during a one-month period: professional contact; physical symptoms; psychosocial problems; prescribing; and care planning.	HIV	Yes	Yes	Yes	Yes	No	No		Age and gender were not predictive of palliative care problems<br>CD4 (b=-0.140, p=0.001) antiretroviral use (b=-0.427, p=0.009) were significantly negative predictors of palliative care problems	5
132.	Kamau et al [111]	2007	Kenya	Quantitative<br>Effect of diagnosis and treatment of inoperable cervical cancer on quality of life among women receiving radiotherapy at Kenyatta National Hospital	Cervical Cancer	Yes	Yes	Yes	No	Yes	No		Perception of availability of social support was significantly lower (p<0.05) among age 50 and above (71.4%) compared to less than 50yrs (56.1%)<br>Interest in coitus was significantly lower (p<0.001) among age 50 and above (4.4%) compared to less than 50yrs (24.3%)	4

133.	Mutimura et al [117]	2007	Rwanda	Quantitative examined the relationship between Body Fat Redistribution and QoL in HAART-treated HIV+ African men and women with BFR in Rwanda.	HIV	Yes	Yes	Yes	No	No	No		Patients with BFR had significantly lower scores on psychological (11.0±2.4 vs 17.1±5.8 p<0.001) and social relationship (9.0±3.2 vs 17.8±5.1, p<0.0001) domain of QOL but no significant difference in overall QOL and physical and independence domains. Women with BFR reported less satisfaction with psychological wellbeing and social relationships.	3
134.	Voss et al [129]	2007	Botswana Lesotho South Africa Swaziland	Quantitative to describe variables that contribute to the differences in fatigue severity and identify predictors and correlates with regard to demographic, HIV disease, and symptom variables.	HIV/AIDS	Yes	Yes	No	No	No	No		Fatigue was significantly increased by inadequate income (t=-3.185, df 536 p<0.02) and inadequate health insurance coverage (F=5.143, df 2508, p<0.006), increasing number of children (t=1.987, df 536, p<0.047), being from Swaziland (F4.597, df 3530, p<0.003). Personal and environmental factors did not explain any individual variance in the fatigue. Individual predictors that explained variance in fatigue were AIDS diagnosis 4%, severity of fever 3%, gastrointestinal 2%,	4

													depressive 4% and numbness 2% symptoms	
135.	Ssengonzi [89]	2007	Uganda	Qualitative<br>describes the challenges faced by elderly persons (50 years and above) in Uganda, as parents and/or relatives of persons infected by HIV and as caregivers of the infected relatives and their uninfected children.	HIV/AIDS	Yes	Yes	Yes		Yes	Yes	financial impact of illness<br>Nutritional impact of illness<br>Challenges of food security<br>Older carers were developing physical symptoms such as back pain, chest pain and backache from lifting and washing patients		5
136.	Bowie et al [176]	2006	Malawi	Quantitative<br>To provide details of the frequency and severity of common symptom among HIV patients	HIV	Yes	No	No	No	No	No			4
137.	Els et al [115]	2006	South Africa	Quantitative<br>to compare pain control practices for terminally ill patients with HIV and patients with cancer.	HIV<br>Cancer	Yes	No	No	No	No	No		More cancer patients received morphine for symptom management at five days before death (69%vs10%, p<0.001) and on day of their death (86% vs 59%, p<0.038)	5
138.	Kaharuza et al [105]	2006	Uganda	Quantitative<br>To better understand the relationship between socio-demographic factors, CD4 cell count and depressive symptoms	HIV<br>Clinically eligible for ART	No	Yes	No	No	No	No		Depression was associated with female gender (OR 1.62, CI 1.13–2.31, p= 0.008), age greater than 50 years (OR 1.93, CI 1.09–3.42, p= 0.024), no education compared with post-primary education (OR 1.69, CI 1.12–2.52, p = 0.011) and dependent income compared with trade (OR 1.81, CI 1.24–2.66, p= 0.002).<br>The strongest predictor of depressive symptoms was lower CD4 cell count.	5

139.	Demmer [71]	2006	South Africa	Qualitative<br>to give a voice to family AIDS caregivers in South Africa and to shed light on their experience living in a context that is vastly different from those of family AIDS caregivers in the United States and other developed countries.	HIV/AIDS	No	Yes	Yes	Yes	Yes	Yes	Practical help for family caregiver in chores<br>Borrowing money and food<br>Silence and reluctance to talk about the disease indicates information needs which builds fear in patients and anger in caregivers and lack of preparation for death<br>Church playing a role in perpetuation of stigma		5
140.	Iwelunmor et al [87]	2006	South Africa	Qualitative<br>examines the role of family in the care and support of people living with HIV/AIDS (PLWHA) as a way of reducing the burden of stigma in the family.	HIV/AIDS	No	Yes	Yes	Yes	Yes	No	Financial difficulty from los of grant<br>Family as a source of psychological, social and existential support		5
141.	Ahmed et al [72]	2005	Sudan	Quantitative<br>to study the efficacy of different palliative procedures used for symptoms control, degree of patient satisfaction with treatment and quality of life	Breast Cancer	Yes	Yes	Yes	No	Yes	Yes		65% of patients came from rural areas (n=59) . Educational and financial status showed 82% illiteracy (n=74) and 58% low financial state, namely, seeking support for treatment.<br><br>Mood disturbances were significantly more common in those patients who knew the diagnosis compared to the others 26 patients vs 8 ($p < 0.0007$) .	4
142.	Moosa et al. [177]	2005	South Africa	Quantitative<br>to determine the occurrence of depression among HIV-positive patients using the Beck's Depression Inventory (BDI) and to determine a relationship, if any, between depressive symptoms and CD4 count.	HIV	Yes	Yes	No	No	No	No		No correlation between BDI scores and CD4 counts<br>No significant difference in CD4 counts, age, gender, marital status and employment status between patients with	3

													BDI of 10 and bove and those below 10	
143.	Ngoma, T [79]	2005	Tanzania	Quantitative<br>To identify family caregivers needs	HIV/AIDS	No	No	Yes	No	Yes	Yes			2
144.	Shawn et al [178]	2005	South Africa	Quantitative<br>to describe the frequency and severity of symptoms as well as the physical discomfort and psychological distress associated with those symptoms in a rural South African HIV-positive population	HIV	Yes	Yes	Yes	No	Yes	No			4
145.	Cameron et al [179]	2004	South Africa	Quantitative<br>To document the use of sedation for refractory symptoms in patients admitted to an independent palliative care unit.	Unspecified	Yes	No	No	No	No	No			4
146.	Norval [180]	2004	South Africa	Quantitative<br>Symptoms and sites of pain experienced by AIDS patients	AIDS	Yes	No	No	No	No	No			2
147.	Kikule [181]	2003	Uganda	Quantitative<br>To identify the palliative care needs of terminally ill people in Uganda.	Cancer<br>HIV/AIDS	Yes	Can't tell	Yes	No	Yes	No			5
148.	Sepulveda et al [73]	2003	Botswana<br>Ethiopia<br>Tanzania<br>Uganda<br>Zimbabwe	Quantitative<br>to identify the needs of patients and their families,	HIV / AIDS<br>Cancer<br>Unspecified terminal diseases	Yes	Yes	Yes	Yes	Yes	Yes			3
149.	Grant et al [74]	2003	Kenya	Qualitative<br>What constitutes a good death in sub-Saharan Africa?	Cancer<br>HIV/AIDS	Yes	Yes	Yes	Yes	Yes	Yes	Becoming a financial burden<br>Financial needs<br>It is better to know than stay disturbed<br>Spiritual cause of illness		6
150.	Lindsey et al [75]	2003	Botswana	Qualitative<br>To gain a greater understanding of the issues and concerns of family caregivers providing care at home.	AIDS<br>Other terminal illness	Yes	Yes	Yes	Yes	Yes	Yes	Caregivers feeling overwhelmed<br>indicate need for practical support<br>Malnourishment<br>Loss of income and poverty<br>Majority of caregivers reported their quality of life to be poor		6

												Exhaustion (physically and emotionally)<br>Caregivers ensure everyone else is fed with the little food available and neglect themselves<br>Disintegrating extended family		
151.	Murray et al [82]	2003	Kenya	Qualitative<br>To describe the experiences of illness and needs and use of services in two groups of patients with incurable cancer, one in a developed country and the other in a developing country.	Incurable cancer	Yes	Yes	Yes	Yes	Yes	No	Becoming financial burden<br>Money is being spent on me instead of school fees<br>emotional support lacking in the hospital		5
152.	Beck et al [101]	2001	South Africa	Quantitative<br>to document the prevalence and patterns of cancer pain management in the Republic of South Africa.	Cancer	Yes	Yes	Yes	No	No	No		Patients with pain were significantly younger than those without pain (52.2 vs 57.3, p=0.008)<br>More none whites 85% experienced worst pain than whites (65%) p<0.001	5
153.	Uys [76]	2001	South Africa	Qualitative<br>describes the post-implementation evaluation of this model.	HIV/AIDS	Yes	Yes	Yes	No	Yes	Yes	Food<br>Grants<br>Housing<br>Helplessness as they were denied help by healthcare professionals (HCPs)		4
154.	Fainsinger et al [182]	2000	South Africa	Quantitative<br>Examined decisions to use sedation in terminally ill patients.	Cancer<br>Unknown<br>AIDS<br>Non Cancer	Yes	No	No	No	No	No			3
155.	Ndaba-Mbata et al [77]	2000	Botswana	Qualitative<br>investigated what knowledge, information and skills families possessed in regard to the provision of care for their ill relatives in the home.	unspecified terminal illness	Yes	Yes	Yes	No	No	Yes	Homecare as isolating experience for caregivers<br>Carers not being informed of patient's diagnosis when it is infectious<br>Information on how to manage symptoms<br>Mentioned patients' physical symptoms		5

156.	Fainsinger et al [183]	1998	South Africa	Quantitative<br>To describe symptoms at the end of life that had required a sedating management approach.	Cancer<br>HIV<br>Unknown	Yes	No	No	No	No	No			4
157.	Keogh et al [80]	1994	Rwanda	Quantitative<br>identifies the social services and counseling needs of women who are already infected with the HIV virus,	HIV/AIDS	No	No	Yes	No	Yes	Yes			4
158.	Seeley et al [81]	1993	Uganda	Quantitative<br>To examine the assumption that the extended family in Africa provides a safety net for individuals in times of need using data on the care of people With AIDS in a rural population in West Uganda	AIDS	Yes	No	Yes	Yes	Yes	Yes			
159.	Mtalane et al [78]	1993	South Africa	Qualitative<br>What are the experiences of terminal illness among Zulu speaking patients, their families and the caregivers who attend them?”	Cancer<br>Liver Cirrhosis	No	Yes	Yes	Yes	Yes	Yes	Financial help<br>Including family needs as part of care<br>Sensitivity to witchcraft as cause<br>Importance of traditional rites of passage for the dead		5

*Highest appraisal score is 6 except otherwise indicated for mixed methods design
