# Supplementary material for: What constitutes a palliative care need in people with serious illnesses across Africa? A mixed-methods systematic review of the concept and evidence
Source: Palliat Med. 2021 Apr 16;35(6):1052–70. doi: 10.1177/02692163211008784 (PMC8371282; doi:10.1177/02692163211008784)
Supplement: sj-pdf-2-pmj-10.1177_02692163211008784 – Supplemental material for What constitutes a palliative care need in people with serious illnesses across Africa? A mixed-methods systematic review of the concept and evidence [file sj-pdf-2-pmj-10.1177_02692163211008784.pdf]

Supplementary file 2: Search terms

MEDLINE, EMBASE, PSYCINFO, GLOBAL HEALTH and CINAHL

**Palliative care**	
1.	exp Palliative Care/
2	palliative care.mp. [mp=title, abstract, heading word, drug trade name, original title, device manufacturer, drug manufacturer, device trade name, keyword, floating subheading word, candidate term word]
3	palliative therapy.mp. [mp=title, abstract, heading word, drug trade name, original title, device manufacturer, drug manufacturer, device trade name, keyword, floating subheading word, candidate term word]
4	end of life care.mp. [mp=title, abstract, heading word, drug trade name, original title, device manufacturer, drug manufacturer, device trade name, keyword, floating subheading word, candidate term word]
5	exp Terminal Care/
6	terminal care.mp. [mp=title, abstract, heading word, drug trade name, original title, device manufacturer, drug manufacturer, device trade name, keyword, floating subheading word, candidate term word]
7	supportive care.mp. [mp=title, abstract, heading word, drug trade name, original title, device manufacturer, drug manufacturer, device trade name, keyword, floating subheading word, candidate term word]
8	palliat* therapy.mp. [mp=title, abstract, heading word, drug trade name, original title, device manufacturer, drug manufacturer, device trade name, keyword, floating subheading word, candidate term word]
9	hospice care.mp. [mp=title, abstract, heading word, drug trade name, original title, device manufacturer, drug manufacturer, device trade name, keyword, floating subheading word, candidate term word]
10	exp hospice care/
11	symptom* treatment.mp. [mp=title, abstract, heading word, drug trade name, original title, device manufacturer, drug manufacturer, device trade name, keyword, floating subheading word, candidate term word]
12	symptomatic treatment.mp.
13	palliat* treat*.mp. [mp=title, abstract, heading word, drug trade name, original title, device manufacturer, drug manufacturer, device trade name, keyword, floating subheading word, candidate term word]
14	EOL care.mp. [mp=title, abstract, heading word, drug trade name, original title, device manufacturer, drug manufacturer, device trade name, keyword, floating subheading word, candidate term wo
**Serious illnesses**	
15	progressive illness$.mp.
16	life limiting illness$.mp.
17	serious illness$.mp.
18	exp chronic disease/
19	chronic illness$.mp.
20	incurable illness$.mp.
21	life threatening illness$.mp.

22	exp Neoplasms/
23	exp Pulmonary Disease, Chronic Obstructive/
24	life limiting illness*.mp. [mp=title, abstract, heading word, drug trade name, original title, device manufacturer, drug manufacturer, device trade name, keyword, floating subheading word, candidate term word]
25	exp Chronic Disease/
26	chronic illness*.mp. [mp=title, abstract, heading word, drug trade name, original title, device manufacturer, drug manufacturer, device trade name, keyword, floating subheading word, candidate term word]
27	exp Terminally Ill/
28	terminal illness*.mp. [mp=title, abstract, heading word, drug trade name, original title, device manufacturer, drug manufacturer, device trade name, keyword, floating subheading word, candidate term word]
29	chronic diseas*.mp. [mp=title, abstract, heading word, drug trade name, original title, device manufacturer, drug manufacturer, device trade name, keyword, floating subheading word, candidate term word]
30	cancer.mp. [mp=title, abstract, heading word, drug trade name, original title, device manufacturer, drug manufacturer, device trade name, keyword, floating subheading word, candidate term word]
31	neoplasm.mp. [mp=title, abstract, heading word, drug trade name, original title, device manufacturer, drug manufacturer, device trade name, keyword, floating subheading word, candidate term word]
32	exp Heart Failure/
33	heart failure.mp. [mp=title, abstract, heading word, drug trade name, original title, device manufacturer, drug manufacturer, device trade name, keyword, floating subheading word, candidate term word]
34	exp Liver Failure/
35	liver failure.mp. [mp=title, abstract, heading word, drug trade name, original title, device manufacturer, drug manufacturer, device trade name, keyword, floating subheading word, candidate term word]
36	exp Renal Insufficiency/
37	renal failure.mp. [mp=title, abstract, heading word, drug trade name, original title, device manufacturer, drug manufacturer, device trade name, keyword, floating subheading word, candidate term word]
38	exp Kidney Failure, Chronic/
39	end stage renal disease.mp. [mp=title, abstract, heading word, drug trade name, original title, device manufacturer, drug manufacturer, device trade name, keyword, floating subheading word, candidate term word]
49	dementia.mp. [mp=title, abstract, heading word, drug trade name, original title, device manufacturer, drug manufacturer, device trade name, keyword, floating subheading word, candidate term word]Advanced
50	exp dementia/
51	exp Human immunodeficiency virus/
52	HIV.mp. [mp=title, abstract, heading word, drug trade name, original title, device manufacturer, drug manufacturer, device trade name, keyword, floating subheading word, candidate term word]
	AIDS,acquired immunedeficiency syndrome, acquired immunodeficiency
53	stroke.mp. [mp=title, abstract, heading word, drug trade name, original title, device manufacturer, drug manufacturer, device trade name, keyword, floating subheading word, candidate term word]
54	exp cerebrovascular accident/

55	cerebrovascular accident.mp. [mp=title, abstract, heading word, drug trade name, original title, device manufacturer, drug manufacturer, device trade name, keyword, floating subheading word, candidate term word]
56	neurologic* disease*.mp. [mp=title, abstract, heading word, drug trade name, original title, device manufacturer, drug manufacturer, device trade name, keyword, floating subheading word, candidate term word]
57	exp neurologic disease/
58	exp multiple sclerosis/
59	multiple sclerosis.mp. [mp=title, abstract, heading word, drug trade name, original title, device manufacturer, drug manufacturer, device trade name, keyword, floating subheading word, candidate term word]
**Africa**	
40	Africa.mp. [mp=title, abstract, heading word, drug trade name, original title, device manufacturer, drug manufacturer, device trade name, keyword, floating subheading word, candidate term word]
41	exp AFRICA/
42	exp AFRICA, EASTERN/
43	exp AFRICA, NORTHERN/
44	exp "AFRICA SOUTH OF THE SAHARA"/
45	exp AFRICA, SOUTHERN/
46	exp AFRICA, WESTERN/
47	exp SOUTH AFRICA/
48	exp AFRICA, CENTRAL/

SCOPUS

((TITLE-ABS-KEY (palliative AND care)) OR (TITLE-ABS-KEY(end AND of AND life AND care)) OR (TITLE-ABS-KEY (terminal AND care)) OR (TITLE-ABS-KEY (supportive AND care)) OR (TITLE-ABS-KEY (palliat* AND therap*)) OR (TITLE-ABS-KEY (hospice AND care)) OR (TITLE-ABS-KEY (symptom* AND treatment)) OR (TITLE-ABS-KEY (palliat* AND treat*)) OR (TITLE-ABS-KEY (eol AND care)) OR (TITLE-ABS-KEY (dying W/3 (care OR comfort OR relief OR strateg* OR plan OR intervention OR pain))))

((TITLE-ABS-KEY (progressive AND illness*)) OR (TITLE-ABS-KEY (serious AND illness*)) OR (TITLE-ABS-KEY (chronic AND disease*)) OR (TITLE-ABS-KEY (chronic AND illness*)) OR (TITLE-ABS-KEY (incurable AND illness*)) OR (TITLE-ABS-KEY (life AND threatening AND illness*)) OR (TITLE-ABS-KEY (neoplasm)) OR (TITLE-ABS-KEY (cancer*)) OR (TITLE-ABS-KEY (chronic AND obstructive AND pulmonary AND disease)) OR (TITLE-ABS-KEY (terminally AND ill)) OR (TITLE-ABS-KEY (terminal AND illness*)) OR (TITLE-ABS-KEY (heart AND failure)) OR (TITLE-ABS-KEY (liver AND failure)) OR (TITLE-ABS-KEY (renal AND failure)) OR (TITLE-ABS-KEY (kidney AND failure)) OR (TITLE-ABS-KEY (renal AND insufficiency)) OR (TITLE-ABS-KEY (chronic AND kidney AND failure)) OR (TITLE-ABS-KEY (kidney AND disease)) OR (TITLE-ABS-KEY (end AND stage AND renal AND disease)) OR (TITLE-ABS-KEY (multiple AND sclerosis)) OR (TITLE-ABS-KEY (neurologic* AND disease*)) OR (TITLE-ABS-KEY (cerebrovascular AND accident)) OR (TITLE-ABS-KEY (stroke)) OR (TITLE-ABS-KEY (hiv)) OR (TITLE-ABS-KEY (hiv)) OR (TITLE-ABS-KEY (human AND immun?deficiency AND virus)) OR (TITLE-ABS-KEY (acquired AND immun? AND deficiency AND syndrome)) OR (TITLE-ABS-KEY (aids)) OR (TITLE-ABS-KEY (acquired AND immun?deficiency AND syndrome)) OR (TITLE-ABS-KEY (human AND immun? AND deficiency AND virus)) OR (TITLE-ABS-

KEY (dementia)) OR (TITLE-ABS-KEY (degenerative AND disorder)) OR (TITLE-ABS-KEY (neurodegenerative AND disorder)))

((TITLE-ABS-KEY (africa*)) OR (TITLE-ABS-KEY (subsahara* AND africa*)) OR (TITLE-ABS-KEY (west* AND africa*)) OR (TITLE-ABS-KEY (south* AND africa*)) OR (TITLE-ABS-KEY (east* AND africa*)) OR (TITLE-ABS-KEY (central AND africa*)) OR (TITLE-ABS-KEY (north* AND africa*))))
